# Scale‐Dependent Effects of Plant Diversity Drivers Across Different Grassland Habitats in Ukraine

**DOI:** 10.1002/ece3.70941

**Published:** 2025-02-12

**Authors:** Oksana Buzhdygan, Selina Baldauf, Dariia Borovyk, Denys Vynokurov, Emma Ladouceur, Olha Chusova, Svitlana Iemelianova, Vasyl Budzhak, Britta Tietjen, Olga Bezrodnova, Olesya Bezsmertna, Illya Chorney, Iwona Dembicz, Jürgen Dengler, Yakiv Didukh, Monika Janišová, Oleksandr Khodosovtsev, Oksana Kucher, Ivan Moysiyenko, Alla Tokariuk, Iuliia Vasheniak, Olena Yavorska, Jonathan Chase, Anna Kuzemko

**Affiliations:** ^1^ Freie Universität Berlin, Institute of Biology Theoretical Ecology Berlin Germany; ^2^ M.G. Kholodny Institute of Botany National Academy of Sciences of Ukraine Kyiv Ukraine; ^3^ Department of Botany and Zoology, Faculty of Science Masaryk University Brno Czech Republic; ^4^ Institute of Biology/Geobotany and Botanical Garden Martin Luther University Halle‐Wittenberg Halle Germany; ^5^ German Centre for Integrative Biodiversity Research (iDiv) Halle‐Jena‐Leipzig Leipzig Germany; ^6^ Department of Biology University of Prince Edward Island Charlottetown Prince Edward Island Canada; ^7^ Canadian Centre for Climate Change and Adaptation University of Prince Edward Island St. Peter's Bay Prince Edward Island Canada; ^8^ School of Climate Change and Adaptation University of Prince Edward Island Charlottetown Prince Edward Island Canada; ^9^ Institute for Evolutionary Ecology National Academy of Sciences of Ukraine Kyiv Ukraine; ^10^ Berlin‐Brandenburg Institute of Advanced Biodiversity Research (BBIB) Berlin Germany; ^11^ V.N. Karazin Kharkiv National University Kharkiv Ukraine; ^12^ Taras Shevchenko National University of Kyiv Educational and Scientific Centre “Institute of Biology and Medicine” Kyiv Ukraine; ^13^ Yuriy Fedkovych Chernivtsi National University Chernivtsi Ukraine; ^14^ Faculty of Biology University of Warsaw Warsaw Poland; ^15^ Vegetation Ecology Research Group, Institute of Natural Resource Sciences (IUNR) Zurich University of Applied Sciences (ZHAW) Wädenswil Switzerland; ^16^ Institute of Botany Plant Science and Biodiversity Centre, Slovak Academy of Sciences Banská Bystrica Slovak Republic; ^17^ Kherson State University Ivano‐Frankivsk Ukraine; ^18^ F.E. Falz‐Fein Biosphere Reserve «Askania Nova» Kherson Ukraine; ^19^ Vasyl' Stus Donetsk National University Vinnytsia Ukraine

**Keywords:** **β**‐diversity, biodiversity, biodiversity drivers, fine spatial scale, grasslands, scale‐dependency

## Abstract

Understanding the factors governing grassland biodiversity across different spatial scales is crucial for effective conservation and management. However, most studies focus on single grain sizes, leaving the scale‐dependent mechanisms of biodiversity drivers unclear. We investigated how climate, soil properties, abiotic disturbance, and land use influence plant diversity across two fine spatial scales in various grassland types in Ukraine. Using spatially explicit data on plant species presence and their cover, collected at smaller (10 m^2^) and larger (100 m^2^) grain sizes, we assessed spatial **β**‐diversity—the variability of biodiversity between scales. We analyzed whether the effects of ecological drivers on **β**‐diversity are mediated by changes in species evenness, density (total cover), and intraspecific aggregation in plant community. In our study, the most influential factors of local plant diversity at both grain sizes were climate variables, followed by soil humus content, litter cover, and soil pH. Soil and litter effects were primarily driven by the response of locally rare species, while climate and grazing effects were driven by locally common species. The strength of most of these effects varied between spatial scales, affecting **β**‐diversity. Soil properties influenced **β**‐diversity through changes in total plant community cover, while the effects of climate and litter operated via changes in species evenness and aggregation. Our findings highlight that biodiversity responses to climate, soil factors, and litter depend on the size of the sampled area and reveal the role of total plant cover, evenness, and aggregation in driving fine‐scale **β**‐diversity in grasslands across different habitat types.

## Graphical abstract

Our findings support the idea that the responses of biodiversity to climate, soil factors, and litter depend on the size of the sampled area. We have identified main mechanisms behind the scale‐dependency of biodiversity drivers and highlighted the significance of locally rare and common species. Our results underscore the importance of incorporating the scale‐dependency of biodiversity drivers in conservation efforts, management strategies, and analyses of global change impacts, which would enhance our ability to predict potential biodiversity change.

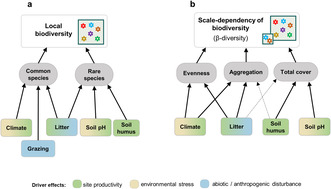

## 1 Introduction

The variability in the numbers of species that occur and persist in a given area, known as biodiversity, remains one of the most well‐studied but poorly understood phenomena in ecological investigation (Díaz and Malhi 2022; Hillebrand et al. 2018). Environmental drivers, such as climate, edaphic factors (Sala et al. 2000; Ulrich et al. 2014), and land use (Díaz et al. 2019; Newbold et al. 2015; Sala et al. 2000), can play a critical role in determining biodiversity. However, differences in the grain size (hereafter scale) at which biodiversity is quantified (Chase et al. 2018; McGill 2010a), as well as the metrics by which it is measured (Chao, Chiu, and Jost 2014; Jost 2006), can dramatically influence our conclusions about the importance of these drivers for biodiversity change (Field et al. 2009; Siefert et al. 2012). The limited understanding of such scale‐dependency of biodiversity drivers and their underlying mechanisms hampers the translation of findings from local plot‐scale to the scales relevant to management, conservation, and restoration policies (Barton et al. 2013; Chase et al. 2019; Ladouceur et al. 2023; Primack et al. 2018; Smith 2010) and impairs our ability to accurately predict biodiversity change and their consequences for ecosystem functions and services (Buzhdygan et al. 2020a).

Spatial variability in the composition of grassland plant communities is particularly high at fine spatial scales (< 100 m^2^) (Biurrun et al. 2021), which are commonly used for sampling grassland vegetation (Chytrý and Otýpková 2003). At the same time, at fine scales, grasslands are remarkably species‐rich and often have even higher plant diversity than tropical forests (Biurrun et al. 2021; Wilson et al. 2012), especially in temperate regions (Dengler et al. 2020). For example, a site in Ukraine had 119 species in 16 m^2^ (Roleček et al. 2019), a site in Romania had 98 species in 10 m^2^ (Wilson et al. 2012), and another grassland site in Ukraine had up to 12 species in 1 cm^2^ (Moysiyenko et al. 2022). Despite their high biodiversity, temperate grasslands are among the most threatened ecosystems due to global change and are among the least protected ecosystems globally (Petermann and Buzhdygan 2021). Understanding the factors that govern grassland biodiversity remains a major challenge in grassland ecology because the responses of grassland plant diversity along natural and anthropogenic gradients highly depend on the spatial scale at which data were collected and analyzed (for details, see Table S1). However, most of this existing evidence is limited to specific grassland types and usually varies in the sampling grain size, thus hindering our ability to test the generality and consistency of scale‐dependency in biodiversity drivers across different grassland habitats (Biurrun et al. 2021). For example, while it is commonly assumed that edaphic drivers dominate at smaller spatial scales, and climate and land use have greater influence at larger scales (Auestad, Rydgren, and Økland 2008; Bergauer et al. 2022; Dembicz et al. 2021b; Kuzemko et al. 2016; Olagoke et al. 2023; Talebi et al. 2021; Turtureanu et al. 2014), some studies contradict these patterns (Chytrý et al. 2015; Polyakova et al. 2016).

Spatial variability in biodiversity is typically quantified by metrics of **β‐**diversity, which link smaller (**α**‐diversity) and larger (**γ**‐diversity) scales, for example, Whittaker's (1972) multiplicative **β‐**diversity (**γ/α**). Scale‐dependent effects of ecological drivers (i.e., effects on **β‐**diversity) at fine spatial scales can be mediated by the following three processes: species density (number of individuals per area), evenness (similarity in the relative abundance among species), and intraspecific aggregation (clustering of conspecifics in spatial distribution) (Blowes et al. 2022; Chase et al. 2018; Chase and Knight 2013; He and Legendre 2002; McGill 2011; Storch 2016; Tjørve et al. 2008). Areas with higher species density within the community generally have greater species richness (*More Individuals Effect*, Srivastava and Lawton 1998), resulting in higher likelihood of species detection at a smaller scale and, thus, in lower spatial variability of biodiversity (Gaston 2000). Similarly, higher species evenness increases richness at smaller spatial scales, thereby reducing differences in species richness across scales (Chase and Knight 2013). In contrast, spatial aggregation reduces richness at smaller scales because aggregated species are less likely to be encountered at a limited area. However, as area increases, the effect of intraspecific aggregation becomes weak due to the higher probability to sample the aggregated species (Chase and Knight 2013). Despite the development of a formal theory that integrates these mechanisms and links them to biodiversity drivers (e.g., McGill 2010b; Chase and Knight 2013; May et al. 2018; Storch, Bohdalková, and Okie 2018), these mechanisms have rarely been tested for different biodiversity drivers in grasslands (e.g., DeMalach et al. 2019). However, such a mechanistic understanding is important because depending on the mechanism through which the biodiversity drivers operate, they can shift the direction of their effects (Bergauer et al. 2022; Kuzemko et al. 2016) or change the shape of the effects with scale (Chase and Leibold 2002; Šímová, Li, and Storch 2013). Furthermore, the role of the responses of locally rare species in these mechanisms remains not clear.

Here, we investigated the potential drivers of plant diversity in grasslands, including climate, soil properties, litter cover, and land‐use management, and disentangled the roles of locally rare and common species in these effects. We examined the scale‐dependency of these drivers and the mechanisms that underlie their effects. For this, we used spatially explicit fine‐scale data on the relative cover of each plant species (vascular and non‐vascular) in the community sampled at two grain sizes (10 m^2^ as the smaller scale and 100 m^2^ as the larger scale) in all grassland habitat types in Ukraine. Such data are strongly underrepresented in international research initiatives, similar to other countries in Eastern Europe, thus limiting our ability to support appropriate management and conservation efforts in these regions (Chytrý et al. 2019). For example, Ukrainian grasslands, which are among the world plant diversity hotspots (Kuzemko et al. 2016; Moysiyenko et al. 2022), are still understudied compared to other grassland regions, especially unique natural grassland habitats, such as Ukrainian Steppes (Borovyk et al. 2023; Kuzemko et al. 2016). Studies of scale‐dependency of biodiversity drivers in Ukrainian grasslands are scarce and focused only on single grassland types (Borovyk et al. 2023; Kuzemko et al. 2016). Closing the geographical gaps for such data would help us to respond more effectively to the global ecological and societal challenges (Chytrý et al. 2019). In this study, we address the following questions: (1) What drives local plant diversity, and do these effects result from the responses of locally rare or common species? (2) Do these effects depend on the sampled grain (i.e., affect **β**‐diversity)? (3) How is the scale‐dependency of biodiversity drivers (the effects on **β**‐diversity) mediated by density (measured by total cover), evenness, and intraspecific aggregation in plant community?

## 2 Materials and Methods

### 2.1 Study Area

**FIGURE 1:**
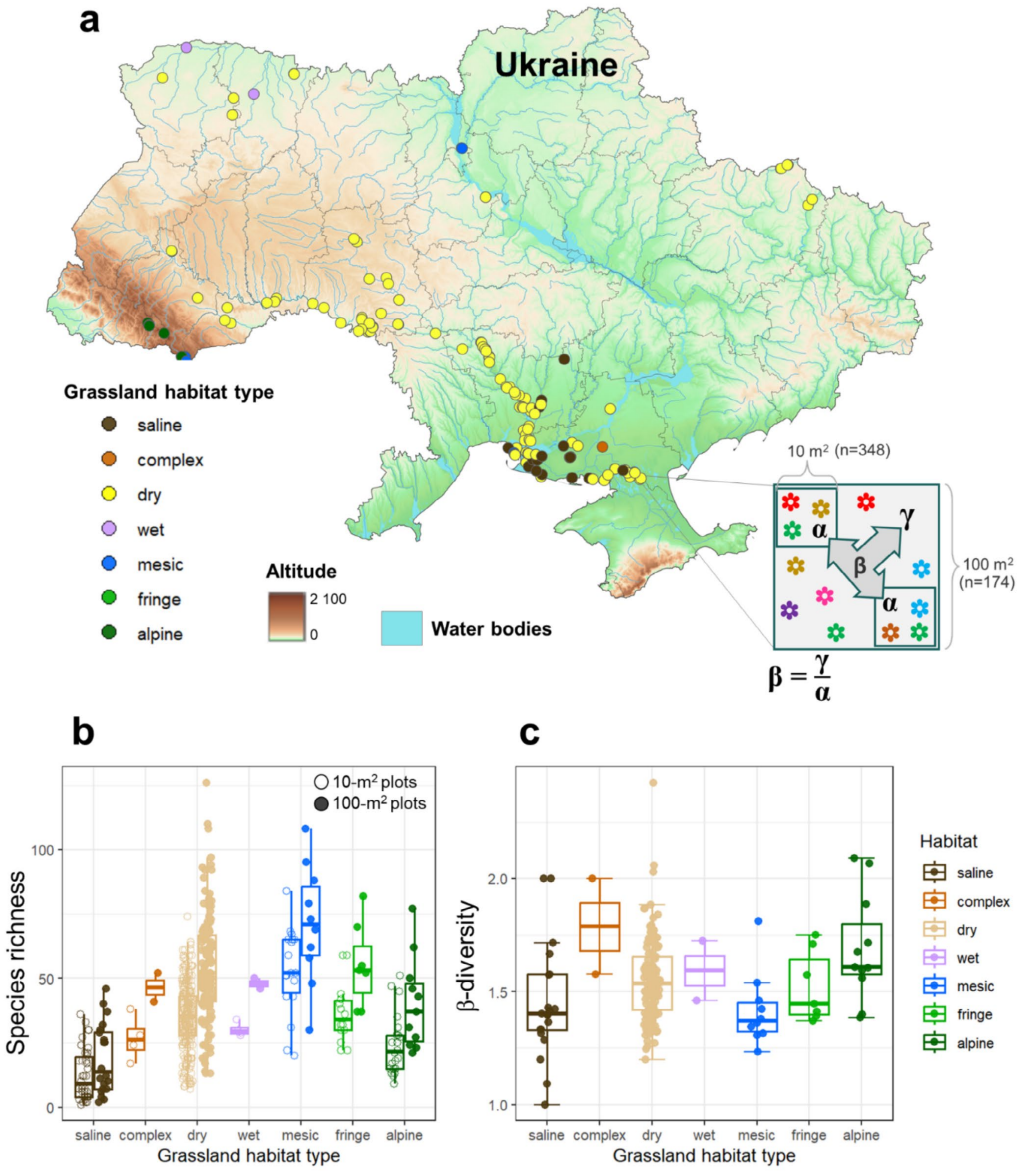
(a) Map of Ukraine showing 174 vegetation plots, indicated with the points on the map (some points overlap), where the color of the points indicates different grassland habitat type. Each of the monitoring plots has grain size 100 m^2^ and is referred to as larger scale, notated by **γ**. Each vegetation plot includes two nested subplots (348 subplots in total) with the grain size 10 m^2^, referred to as smaller scale, notated by **α**. The icons of different colors on the scheme of the nested‐plot design represent different plant species. Beta (**β**) is the scaling factor among the two grain sizes and shows the spatial difference in plant biodiversity (species richness or evenness) between smaller and larger fine spatial scales. The map was created using QGIS software (QGIS Development Team, 2023). (b) Plant species richness for each grain size within each grassland habitat type (indicated by different colors). (c) **β**‐species richness for each grassland habitat type. Boxplot shows minimum, median, and maximum values of species richness. Points show the values for each plot.

We sampled grasslands of all major grassland habitat types of Ukraine (Table S2) – (Kuzemko et al. 2022). All plots were sampled during 2010–2022, resulting in 11 datasets (see Table S2). The geographical extent of the data covered an area from 46.08° N to 51.87° N and 24.2° E to 37.76° E and an elevational gradient from 0 m to 1805 m a.s.l. (Figure 1a).

The average annual temperature in the study area ranges from 0.3°C to 11.4°C (Karger et al. 2018). In the North part of Ukraine, the average January temperature ranges from −3°C to −2°C, while in the South, it ranges from −2°C to −1°C. The average July temperature in the North ranges from +18°C to +21°C, and in the South part of Ukraine, it ranges from +23°C to +25°C. In the Carpathian region, the average annual temperature on the upper belts is about 7°C–10°C. In the mountains, the average January temperature ranges from −10°C to −8°C, and in July, the average temperature at an altitude of 1500 m a.s.l. is about 10°C. The annual precipitation range is 700–800 mm in the Northern part of the study area (Volyn region and Rivne region) and 400–550 mm per year in the South (Mykolaiv and Kherson regions). The Carpathians have significantly higher levels of precipitation, reaching up to 1600 mm at an altitude of 1500–1800 m a.s.l. (Buzhdygan et al. 2020b).

### 2.2 Plot Design, Sampling, and Biodiversity Predictors

In each study grassland, we identified a large vegetation patch that was well representative of the target grassland type, and the study plots were established in this patch. Sampling design was based on the standard sampling methodology (Dengler et al. 2016) of the Eurasian Dry Grassland Group (EDGG, https://edgg.org). Each plot of 100 m^2^ (*n* = 174) included two 10 m^2^ subplots (*n* = 348) situated in opposite corners (Figure 1a). Within each plot and subplot, we recorded all species of vascular plants, terricolous bryophytes, and lichens. Study grasslands were selected to cover all possible grassland habitat types according to the EUNIS system v.2018 (Schaminée et al. 2018). The habitat types were preliminarily identified in the field with subsequent verification using the EUNIS‐ESy expert system at 3rd level of hierarchy (Chytrý et al. 2020). However, for the propose of this study, we assigned these habitat types to the groups which correspond to the 2nd level of the EUNIS hierarchy: dry, mesic, wet, alpine, fringe, and saline. One of the study grasslands—the depressions (called *pody*) of the Steppe zone, is not currently in the EUNIS‐ESy expert system. It was identified based solely on the environmental characteristics (Shapoval and Kuzemko 2021). We classified *pody* as a group of habitat complexes, which are defined as the heterogeneous combinations of different habitat types that can coexist at the same location over time or occur across spatial mosaics (Evans 2016). The taxonomic nomenclature for vascular plants followed Euro+Med (2006+) for bryophytes (Hodgetts et al. 2020) and for lichens (Kondratyuk et al. 2021). For each plant species, we recorded its estimated cover in percent (Dengler and Dembicz 2023).

At each plot, we recorded litter cover, level of grazing intensity, and presence/absence of mowing. In each 10 m^2^ subplot, we took soil samples from the upper 10 cm of the soil surface in five random locations. The level of grazing intensity (ordinal variable with four levels) was estimated in the field ranging from 0—no grazing to—intensive grazing. We measured soil pH electrometrically in a suspension of 5 mL soil with 25 mL deionized water. Soil organic carbon (humus content) was measured for each sample using 0.4 N potassium dichromate solution in accordance with Tyurin's method. Litter cover in grasslands indicates productive communities that develop with moderate disturbances and not extremely harsh environmental conditions (Facelli and Pickett 1991; Grime 1979). However, a major management problem associated with the abandonment of highly productive grasslands is the increase in above‐ground biomass and the subsequent litter accumulation (Ruprecht et al. 2010), which, in excessive amounts, become disturbances to plant community assembly processes (Facelli and Pickett 1991; Ruprecht and Szabó 2012). Therefore, litter cover can be considered as an indication of productivity (high levels) and as a proxy of disturbance at both low litter cover (Dembicz et al. 2021a) and high litter cover (Ruprecht et al. 2010; Ruprecht and Szabó 2012). Land use is considered an anthropogenic disturbance. High and low levels of soil pH represent factors of soil‐related stress to the plant community, as well as soil toxicity to plants at low pH. Soil organic carbon indicates site productivity for the grassland plant community.

For each 100 m^2^ plot, using plot coordinates, we extracted the following climatic variables from the CHELSA climate database (Karger et al. 2018): Mean annual temperature, mean annual precipitation, and precipitation seasonality—the intra‐annual precipitation variation, quantified as the standard deviation of the monthly estimates of precipitation from the annual mean. Annual temperature and precipitation exhibited a strong negative correlation (Figure S1a). To derive a single composite variable of climate gradient of mean annual precipitation and temperature, we first centered the temperature and precipitation using the *scale* function in R version 4.2.2 (R Core Team 2022) and then performed a principal component analysis (PCA) using the *prcomp* function. The first principal component explained 98% of the variance and correlated positively with increasing precipitation and decreasing temperature, representing a gradient ranging from hot and dry to cold and wet climatic conditions (Figure S11). This first principal component was used as a single variable representing climate gradient of mean annual precipitation and temperature in our analysis (hereafter, climate gradient).

### 2.3 Biodiversity Measures

We assessed plant diversity at two spatial scales: 10 m^2^ plots (*n* = 348) and 100 m^2^ plots (*n* = 174). Plant diversity was assessed for the entire community, including vascular plants, terricolous bryophytes, and lichens. At each scale, we calculated species richness, representing the number of plant species recorded. Additionally, as a measure of community evenness, we calculated ENS_PIE_ (Chase and Knight 2013), using *vegan* packages in R (A. J. Oksanen et al. 2018):

(1) $${\mathrm{ENS}}_{\mathrm{PIE}}=1/\sum_{i=1}^{S}{\;p}_{i}^{2},$$

where *S* is the number of species and $p_{i}$ is the proportion of the community represented by species *i* (Chase and Knight 2013; Jost 2006). The proportion of each plant species ($p_{i}$) was measured by its cover relative to the cumulative cover of the plant community. ENS_PIE_ has been also known as the Hill–Simpson index (Roswell, Dushoff, and Winfree 2021) and is equivalent to the inverse of the traditional Simpson index (Chao, Chiu, and Jost 2014; Roswell, Dushoff, and Winfree 2021). For our study, we chose to use ENS_PIE_ over other known evenness measures because it is directly comparable to species richness and explicitly accounts for the fact that rare species have a disproportionate effect on the measure of species richness (Chase and Knight 2013). Species richness gives high leverage to locally rare species and thus weights rare and common species equally. ENS_PIE_, on the other hand, uses a reciprocal scale, which shifts leverage toward common species, making it dominated by their relative abundance (Roswell, Dushoff, and Winfree 2021). By comparing the responses of species richness to ENS_PIE_, we assessed whether the effects of biodiversity drivers are due to the responses of common or rare species (Ladouceur et al. 2023; Roswell, Dushoff, and Winfree 2021).

We calculated the multiplicative **β**‐diversity metric (Whittaker 1972) as a measure of scale‐dependency of biodiversity:

(2) $$\beta_{i}=\frac{\gamma_{i}}{{\bar{\alpha }}_{i}},$$

where $\beta_{i}$ represents the scaling factor between the two grain sizes, i.e., the spatial difference in biodiversity (species richness or ENS_PIE_) for plot *i*. Here, $\gamma_{i}$ is the biodiversity at the 100 m^2^ scale for plot *i*, and ${\bar{\alpha }}_{i}$ is the mean biodiversity of the two 10 m^2^ subplots nested within the 100 m^2^ plot *i*. We use here the notations **α** and **γ** without making any assumptions about their relationship with local or regional coexistence mechanisms.

### 2.4 Data Analysis

All analyses were carried out in R version 4.2.2 (R Core Team 2022). To test the drivers of plant diversity across scales, we applied linear (LMM) or generalized linear mixed effect models (GLMM), depending on the nature of the response variables. Specifically, for the analysis of species richness at 10 m^2^ scale, we applied GLMM with the Poisson family, using the *glmer* function of the *lme4* package (Bates et al. 2015). For the analysis of species richness at 100 m^2^, scale we first applied GLMM with the Poisson family, but due to overdispersion, we applied the negative binomial family using the *glmer.nb* function from the *lme4* package. For the analysis of beta species richness and for the ENS_PIE_ at all spatial scales (i.e., 10, 100 m^2^, and **β**‐ENS_PIE_), we applied LMM using the *lmer* function from the *lme4* package. The ENS_PIE_ values for all scales were log‐transformed to meet the assumptions of homoscedasticity. Plot ID, nested in the dataset ID, was included as a random effect in all models for the 10 m^2^ scale, and the dataset ID was used as a random effect in 100 m^2^ and in the models for **β**‐diversity, in order to account for the potential similarities in data collected during the same year or sampling campaign (Table S2). We tested random effects in all models and found them to be statistically significant and explaining substantial amounts of data variance. For an overview of the datasets used in this study, see Table S2.

We used a two‐step approach to test the effects of plant diversity drivers. The first model included the following predictors: climate gradient, soil organic carbon, soil pH, litter cover, grazing intensity, and mowing. After inspecting the data, a quadratic term was allocated to climate gradient, soil organic carbon, soil pH, and litter cover to properly model nonlinear responses. Thus, we developed a set of a priori models that allowed for unimodal effects of these predictors in all possible combinations (with and without unimodal effects) and tested if the quadratic terms impacted the predictive ability of the model. For this, we used the Akaike information criterion (AIC) approach to select the most parsimonious model within the 2 units of AIC of the model with the lowest AIC. Precipitation variability was not included in the first model to avoid risk of losing signal in the climate gradient effect on biodiversity due to the co‐variation of precipitation variability with the climate gradient (Figure S1b). Specifically, precipitation variability had the hump‐shaped relationship with climate gradient (Figure S1b), indicating high precipitation seasonality in warm and moderately wet habitats in the middle of the climate gradient (i.e., in mesic and fringe grasslands) and low precipitation variability in dry and hot conditions (i.e., saline, dry, and complex grassland types) and in cold and humid habitats (i.e., alpine grasslands). The effects of precipitation variability were examined separately in the second model, where all predictors (including climate gradient) were fitted as covariates. AIC was again used to evaluate the unimodal effects of precipitation variability. Furthermore, we tested whether precipitation variability adds explanatory power beyond the nonlinear effect of the climate gradient. For this, we compared two models: one with both linear and quadratic terms for the climate gradient and another where the quadratic term was replaced by precipitation variability. We used AIC to compare the fit of these models (Table S6). If the model with precipitation variability had an AIC at least 2 units smaller than the model with the quadratic term of climate gradient, this would provide evidence that the precipitation variability better explains the observed patterns compared to the nonlinear climate gradient alone.

We tested the spatial autocorrelation of the residuals for each model using Moran's I statistics. For this, for each (G)LMM model, we extracted randomized residuals using the *simulateResiduals* function of *DHARMa* package in R (Hartig 2022). Then, on these residuals, we performed Moran's I test using the *testSpatialAutocorrelation* function of *DHARMa* package. The spatial matrix of weights for Moran's I test was calculated (using the *dist* function in R) as the inverse distance matrix (Euclidean distances between pairs of plots) based on longitude and latitude of each study plot. The calculated Moran's I statistics revealed no significant autocorrelation of residuals for any of the models (i.e., as all *p* > 0.05, Tables S3–S5), indicating that spatial autocorrelation among the study plots did not affect our results.

To be able to interpret and compare the parameter estimates on a comparable scale, we have standardized the obtained estimate coefficients (for details, see Supporting Information: Methods). We also compared the relative variance explained by each driver on each sampling scale (Figure 3c) by calculating partial *R* ^2^ from (G)LMMs using the *r2beta* function from the *r2glmm* package in R (Jaeger 2017).

### 2.5 Mediating Drivers of β‐Diversity

**FIGURE 2:**
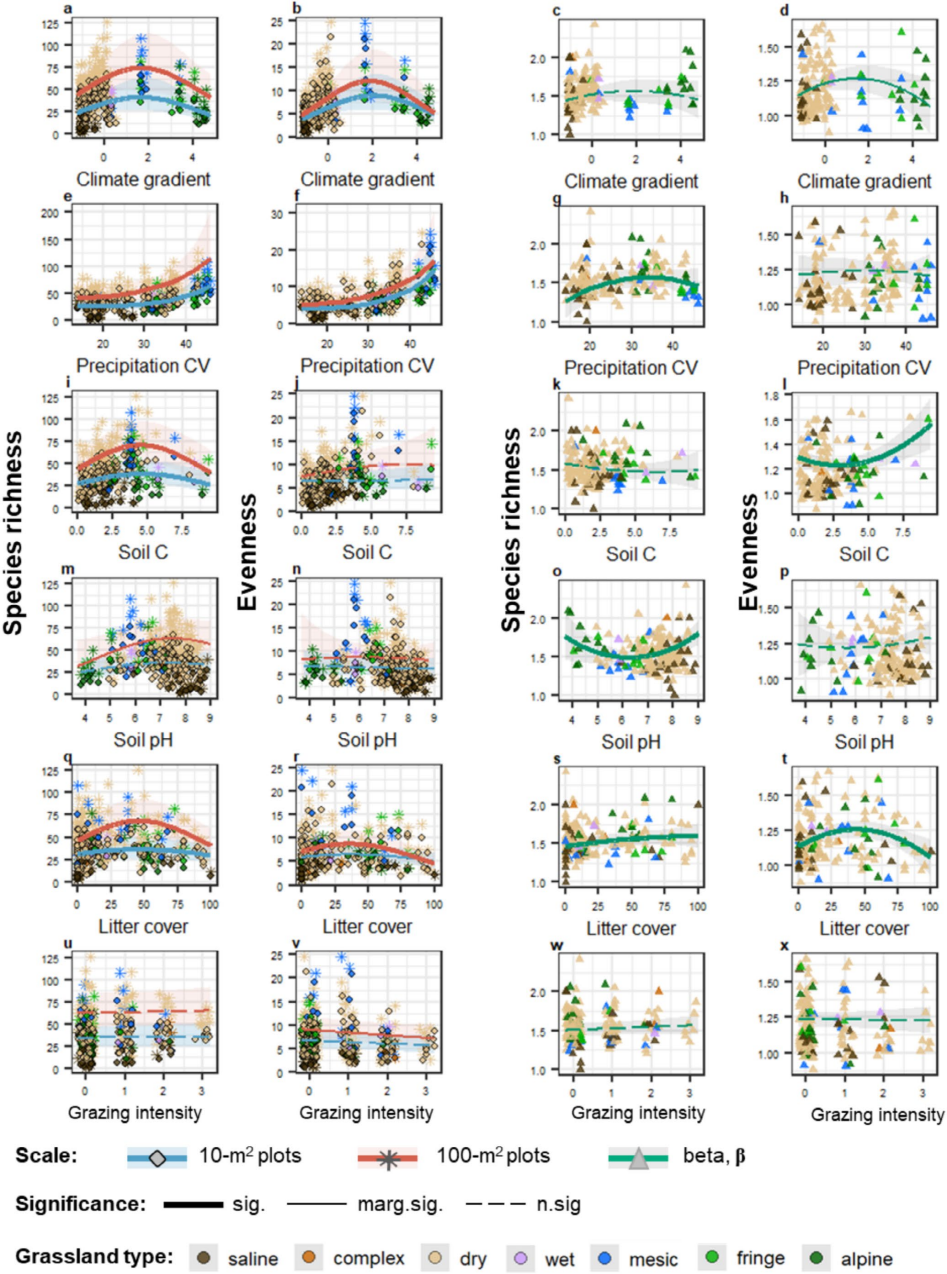
Results from mixed models testing the effects of environmental drivers on species richness and evenness at the 10 and 100 m^2^ plots (shown by blue and red lines, respectively) and on **β‐**diversity—the scaling factor among the two fine‐grain sizes (shown by green lines). For the model results see Tables S3 and S4. Solid thick lines show significant effects (*p* < 0.05), solid thin lines show marginally significant effects (0.05 ≤ *p* ≤ 0.09), and dashed lines show nonsignificant effects (*p* > 0.09). Shaded areas around lines show 95% confidence intervals. Different shapes of data points indicate different spatial scales: diamonds for the 10 m^2^ plots, stars for the 100 m^2^ plots, and triangles for **β‐**diversity. Colors of data points indicate grassland habitat types. To improve the visibility of comparisons among the diversity slopes in 10 and in 100 m^2^, the results for both scales are shown on the same plots. Plots for each scale separately are provided in Figure S5.

In accordance with the theoretical predictions, we considered species density, evenness, and intraspecific aggregation as proximate factors mediating the effects of ecological drivers on **β‐**diversity (Blowes et al. 2022; Chase et al. 2018; Chase and Knight 2013; He and Legendre 2002; McGill 2011; Storch 2016; Tjørve et al. 2008). As a proxy of density, we used total cumulative cover of plant community, measured as the sum of the cover of all species. We did not measure the number of individuals for each species per area; therefore, plant cover served as the best available proxy for density, as used in previous studies (DeMalach et al. 2019; Ladouceur et al. 2023). We used ENS_PIE_ (see Equation 1) as a measure of community evenness. Spatial intraspecific aggregation was estimated by comparing dissimilarity in species covers between the two corners (i.e., two 10 m^2^ plots) within each 100 m^2^ plot. For this, we calculated the balanced variation component of Bray–Curtis dissimilarity in species cover using *betapart* package in R (Baselga and Orme 2012). This measure is independent of total community abundance (total plant cover in our study) and measures the balanced variation in species abundance between two quadrats, i.e., when cover increases for some species and decreases for others, maintaining similar total cover across quadrats, including also species turnover, where abundance of one species is replaced by other species (Baselga 2017). Higher dissimilarity in covers of taxa between the two 10 m^2^ corners within the same 100 m^2^ plot implies higher intraspecific aggregation. We tested the effects of these proximate factors (evenness, total cover, and aggregation) on **β‐**diversity (Figure 5). Furthermore, we tested the effects of biodiversity drivers on each of these proximate factors (Figure 2, Figures S8 and S9).

## 3 Results

Overall, we found 1560 taxa (species, subspecies and aggregates) across all the study plots, out of which 1233 species of vascular plants, 171 species of bryophytes, and 156 species of lichens. Species richness increased with the sampled scale (Figure S3, Figure 1b), but the difference among scales depended on the grassland habitat type (Figure 1b,c).

### 3.1 Effects of Climate

Both species richness and ENS_PIE_ at the 10 and 100 m^2^ plots showed hump‐shaped responses to the climate gradient PC (Figure 2a,b). The effects on $\beta_{{\mathrm{ENS}}_{\mathrm{PIE}}}$ were also hump‐shaped but marginally significant, while $\beta_{\text{richness}}$ showed no significant responses (Figure 2c,d). Increased intraannual variation in precipitation led to higher species richness and ENS_PIE_ measures on both scales (Figure 2e,f), with a hump‐shaped effect on $\beta_{\text{richness}}$ but no significant effects on $\beta_{{\mathrm{ENS}}_{\mathrm{PIE}}}$ (Figure 2g,h).

### 3.2 Effects of Soil Properties

**FIGURE 3:**
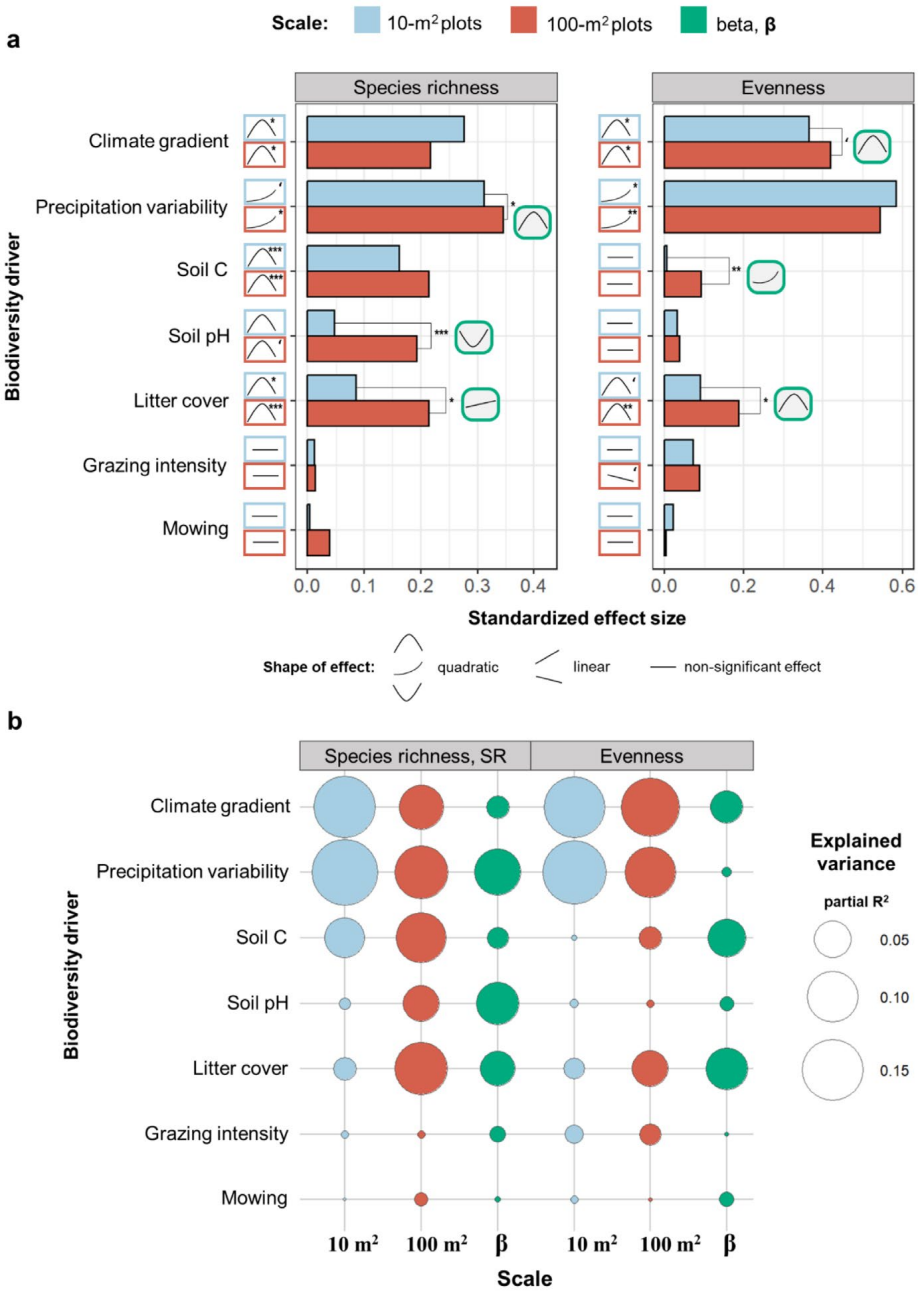
(a) Relative strengths (standardized effect size) of the effects of each environmental driver on local diversity measures (species richness and ENS_PIE_), shown by blue and red bars for the 10 and 100 m^2^ plots, respectively. Blue and red icons show the shape of the effects and their significance for each driver and respective scale, with the following levels of significance: *p < 0.05; **p ≤ 0.01; ***p ≤ 0.001; and ‘*p* ≤ 0.09 (marginally significant). Green icons show the shape for only significant effects (bars marked by stars) on **β‐**diversity—the scaling factor among the two spatial scales. (b) Relative importance of the environmental drivers in governing **β‐**diversity measures and local diversity in 10 and 100 m^2^. Circle sizes are proportional to the fraction of variance explained (partial R^2^) by the study drivers for each response variable. For the model results, see Tables S3 and S4.

At both 10 and 100 m^2^ scales, species richness had a hump‐shaped relationship with soil humus (Figure 2i) and soil pH (Figure 2m), but these effects were more pronounced in 100 m^2^ (Figure 3a,b). However, none of the studied soil properties significantly influenced local‐scale ENS_PIE_ (Figure 2j, Figure 2n). While the soil humus content showed no significant effect on $\beta_{\text{richness}}$, we observed a U‐shaped effect on $\beta_{{\mathrm{ENS}}_{\mathrm{PIE}}}$ (Figure 2k,l). Instead, we found a strong U‐shaped effect of soil pH on $\beta_{\text{richness}}$ but no effects on $\beta_{{\mathrm{ENS}}_{\mathrm{PIE}}}$ (Figure 2o,p).

### 3.3 Effects of Litter Cover

The cover of plant litter had hump‐shaped effects for both species richness and ENS_PIE_ at the 10 and 100 m^2^ plots, with ENS_PIE_ showing notably weaker responses compared to species richness (Figure 2q,r, Figure 3b). Furthermore, the relative importance of litter cover in predicting plant diversity was higher at the 100 m^2^ plots than in 10 m^2^ (Figure 3a,b). The measures of **β**‐diversity had distinct responses to litter cover: Species richness increased with the litter cover, while ENS_PIE_ showed hump‐shaped response (Figure 2s,t).

### 3.4 Effects of Land Use

We detected no significant effects of mowing on any biodiversity measures across the studied spatial scales (Tables S3 and S4, Figure S6). Similarly, grazing intensity did not significantly affect species richness across two local scales (Tables S3 and S4, Figure 2u). However, contrary to species richness, there was a declining trend in ENS_PIE_ in 100 m^2^ with increasing grazing intensity, although the effect was only marginally significant (Tables S3 and S4, Figure 2v).

### 3.5 Total Plant Cover

We found a hump‐shaped relationship between total plant cover and species richness at both grain sizes and a U‐shaped relationship with $\beta_{\text{richness}}$ (Figure S4). Soil humus content, soil pH, and litter cover had curvilinear effects of the total cover of the plant community (Figure S8e–j), although the effects of litter cover were relatively weak, especially on the larger scale (Figure S8j).

### 3.6 Proximate Drivers of β‐Diversity

$\beta_{\text{richness}}$ showed a hump‐shaped relationship with the total plant cover, a negative relationship with evenness, and a positive relationship with species aggregation (Figure 5). The total plant cover was significantly influenced by the soil humus content and pH, with a weaker effect of litter cover (Figure S8). $\beta_{{\mathrm{ENS}}_{\mathrm{PIE}}}$ showed a strong positive relationship with aggregation (Figure 5d). Both the climate gradient and litter cover had hump‐shaped effects on intraspecific aggregation (Figure S9).

## 4 Discussion

We sampled grasslands of different habitat types across Ukraine and examined the effects of environmental drivers on plant diversity in a spatially explicit context by partitioning local diversity into two different grain sizes (10 and 100 m^2^) and by examining the scale‐dependency of diversity drivers by testing their effects on the scaling factor between these two spatial scales (**β‐**diversity). We explored both plant species richness and community evenness (measured by ENS_PIE_), enabling us to uncover if biodiversity drivers operated via responses of locally rare or common species. We also explored if the scale‐dependency of biodiversity and of their drivers are mediated by changes in evenness, total cover, or spatial intraspecific aggregation of plant community (Figure 6).

### 4.1 Drivers of Local Plant Diversity

The climate gradient of mean annual precipitation and temperature was among the most important drivers of local plant diversity at both 10 and 100 m^2^ scales, Figure 3. The hump‐shaped effects of climate on plant diversity (Figure 2a) can be attributed to climatic stress, where the co‐occurrences of species are limited by harsh environmental conditions on the low and high ends of the gradient, i.e., cold areas on the high end and hot areas with drought and associated lack of snow cover during winter on the low end of the climate gradient (Figure S1a). The peak in plant diversity in the middle of the gradient is linked to high site productivity due to warm and moderately wet conditions. Furthermore, in the middle of the climate gradient, plant communities were not only the species richest, but also had more even relative cover (i.e., higher ENS_PIE_, Figure 2b). This may be attributed to the facilitative coexistence of stress‐tolerant species with competitive stress‐intolerant species at intermediate levels of environmental stress (Michalet et al. 2006). The quadratic effects of climate gradient on species richness (but not evenness) were driven by the intra‐annual variability in precipitation (Table S6). Richness increased proportionally to the precipitation variability (Figure 2e), likely because the wider range and variation in intra‐annual precipitation allowed for greater niche space with larger ecological trait differences between species and thus more species with suitable niches (Stein, Gerstner, and Kreft 2014). The climate–biodiversity relationships in our study were strongly affected by the responses of common species, as the shape and strengths of the climate effects was similar between species richness and ENS_PIE_ (Figure 2a,b).

Further, we tested the effects of soil properties (when statistically controlling for climate impact) and found the hump‐shaped effects of both soil humus content and pH on species richness at both spatial scales (Figure 2i, Figure 2m). These effects of soil humus were likely related to site productivity, while the effects of soil pH to the environmental stress gradient (for detailed discussion, see Supporting Information: Discussion: Section 2.1). In contrast to climate, the effects of both soil humus and pH on plant diversity were determined by the responses of locally rare species, as we found no significant effects of these variables on ENS_PIE_ (Figure 2j, Figure 2n). High soil productivity (e.g., with increasing soil humus in our study plots, Supporting Information: Discussion: Section 2.1) generally leads to asymmetric competition among plant species, resulting in lower population densities of initially rare species and their subsequent extinctions (Rajaniemi 2003). However, in severe environmental conditions, such as low and high soil pH, biotic interactions become less important than environmental stress, and only stress‐tolerant species can persist (Michalet et al. 2006). Both acidification and high alkalinity of soil limit the plant species pool to pH‐tolerant specialists (Schuster and Diekmann 2003).

Litter amounts in grasslands is generally linked to the productivity–diversity relationship, as litter production is a function of annual net primary productivity in grasslands (Grime 1979). However, in well‐managed sites, litter cover might be influenced by management practices, which could limit its reliability as an indicator of productivity. While litter cover was only weakly correlated with proxies of site productivity in our study (i.e., with soil humus content and climate gradient, Figure S2c,d), litter can profoundly influence plant community structure through mechanisms beyond productivity effects (for details, see Supporting Infomration: Discussion: Section 2.2). Litter often acts as an abiotic disturbance to grassland plant community (Dembicz et al. 2021a; Ruprecht et al. 2010), and the hump‐shaped effects of litter cover on local species richness observed in our study (Figure 2q) are consistent with the *intermediate disturbance hypothesis*, where moderate disturbance levels reduce interspecific competition, promote occurrences of rare species, and increase species richness. The effects of litter on species richness in our study were determined by the responses of both locally common and rare species, with rare species playing an important role, as indicated by relatively weaker effects of litter on ENS_PIE_ than on richness (Figure 2q,r, Figure 3). Moderate amounts of litter can reduce species competition for light resources by physically separating plants and reducing light availability to dominant competitive species (Facelli and Pickett 1991; Lamb 2008). This creates opportunities for less competitive and locally rare species to establish. Similarly, the litter patches foster microscale variations in nutrient availability, promoting niche differentiation and coexistence of diverse plant species.

Mowing had minor effects on plant diversity in our study (Table S3, Figure S6). Grazing intensity also did not alter plant species number, but it reduced the evenness of the plant community (Figure 2v), indicating that some species became more dominant with increasing grazing intensity, likely these were the grazing‐tolerant species favored by grazing (Buzhdygan et al. 2020b). Previous evidence points toward land use as a major driver of local plant diversity in grasslands (Petermann and Buzhdygan 2021; Sala et al. 2000). The low explanatory power of land use in our study may be attributed to the fact that our study plots were deliberately selected to avoid high‐intensity management. This is because the main focus of our study is on the natural biodiversity drivers across different grassland habitat types. Furthermore, the rates of land‐use abandonment in Ukrainian grasslands, as well as across Europe, have increased in recent years (Buzhdygan et al. 2020b; Enyedi, Ruprecht, and Deák 2008; Petermann and Buzhdygan 2021), leading to a rather short gradient of land‐use intensity in our study. Moreover, nearly 48% of our study plots belong to zonal (natural) vegetation (Figure S7), which, unlike seminatural grasslands, are maintained by natural abiotic and biotic processes and do not require human interventions in natural conditions (Török et al. 2018).

Numerous studies in grasslands have identified soil properties, such as humus content and pH, as the most important drivers of the fine‐scale species richness (Chytrý, Tichý, and Rolecek 2003; Chytrý et al. 2007; Dembicz et al. 2021b; Schuster and Diekmann 2003), while other studies found no effect (Kuzemko et al. 2016; Turtureanu et al. 2014). In our study the effects of macroclimatic variables prevailed over those of the local effects of soil properties, litter cover and land use (Figure 3). Our results also demonstrate that the mechanisms underlying environment–biodiversity relationships depended on ecological driver (Figure 6a), with soil properties and litter cover primarily affecting rare species, while climate and grazing predominantly influenced locally common species.

### 4.2 Scale‐Dependency of Plant Diversity and of Diversity–Environment Relationships

**FIGURE 4:**
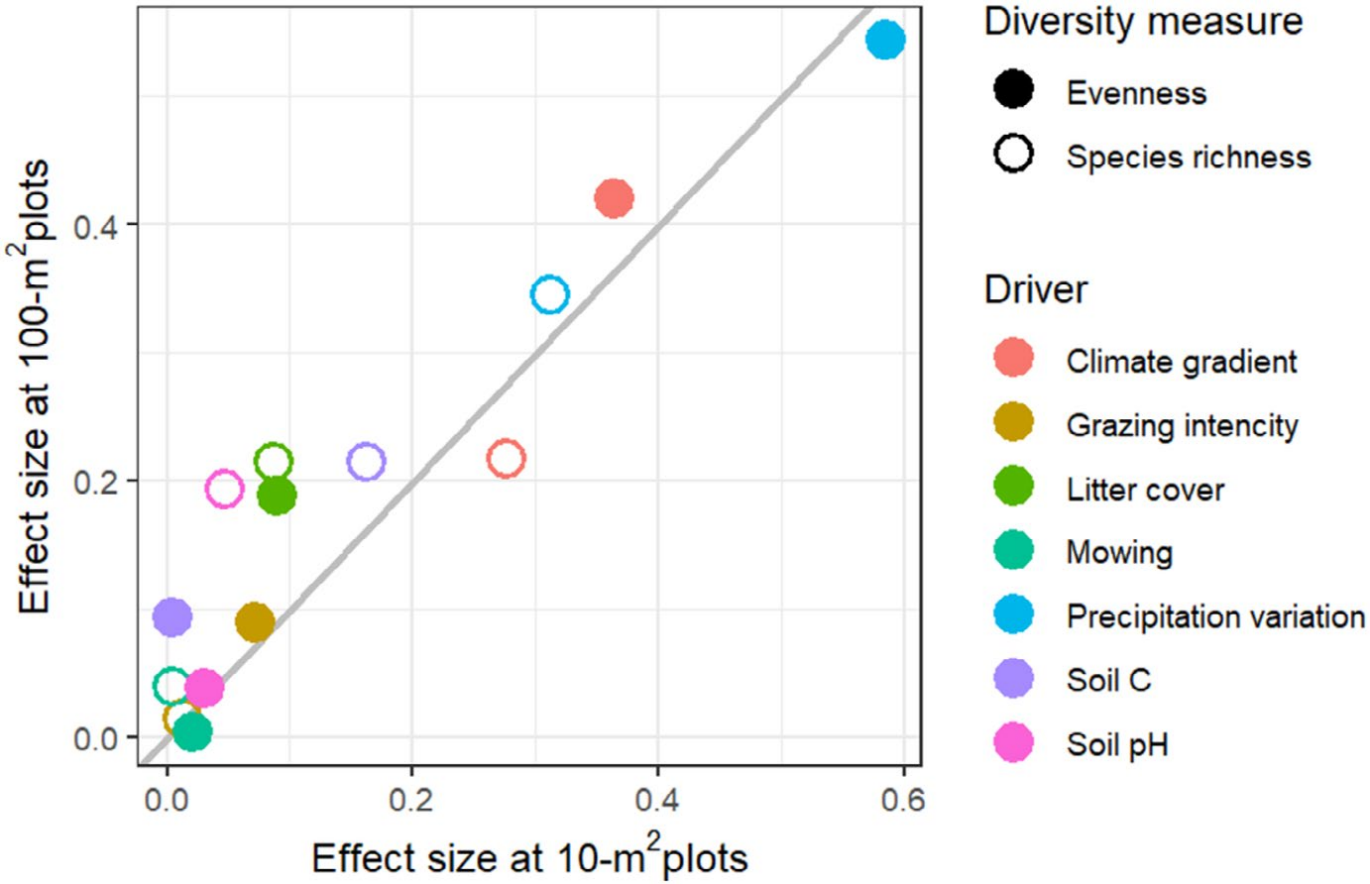
Scale‐dependent effects of environmental drivers on species richness and evenness at small (10 m^2^) and larger (100 m^2^) fine‐grain plots. Points show the standardized effect sizes of each environmental driver (marked by colors of points) on the diversity measures at 10 m^2^ scale (*x*‐axis) compared to 100 m^2^ (*y*‐axis). The solid gray line indicates the 1: 1 line expected if effect sizes were not scale‐dependent. Points above and below this line indicate effect sizes that are larger or smaller, respectively, as grain size increases.

The explanatory power of environmental drivers for plant diversity was weaker at smaller compared to larger grain sizes (Figure S3c,d), consistent with previous studies (Bergauer et al. 2022; Filibeck et al. 2019; Kuzemko et al. 2016; Talebi et al. 2021). This lower predictability is likely due to the greater influence of stochastic processes at finer scales (Barton et al. 2013), which increases variability in species co‐occurrences and thus may weaken the impact of environmental factors on biodiversity. The strength of effects for most biodiversity drivers differed between the two grain sizes (Figures 3 and 4). However, the shape and direction of these effects remained consistent across scales (Figure 2), aligning with most of previous fine‐scale grassland studies (Dembicz et al. 2021a, 2021b; Polyakova et al. 2016; Turtureanu et al. 2014). This pattern contrast with large‐scale studies, such as comparing plot to regional scales, where the shape and direction of effects often change with sampling grain (Chase and Leibold 2002; Šímová, Li, and Storch 2013).

**FIGURE 5:**
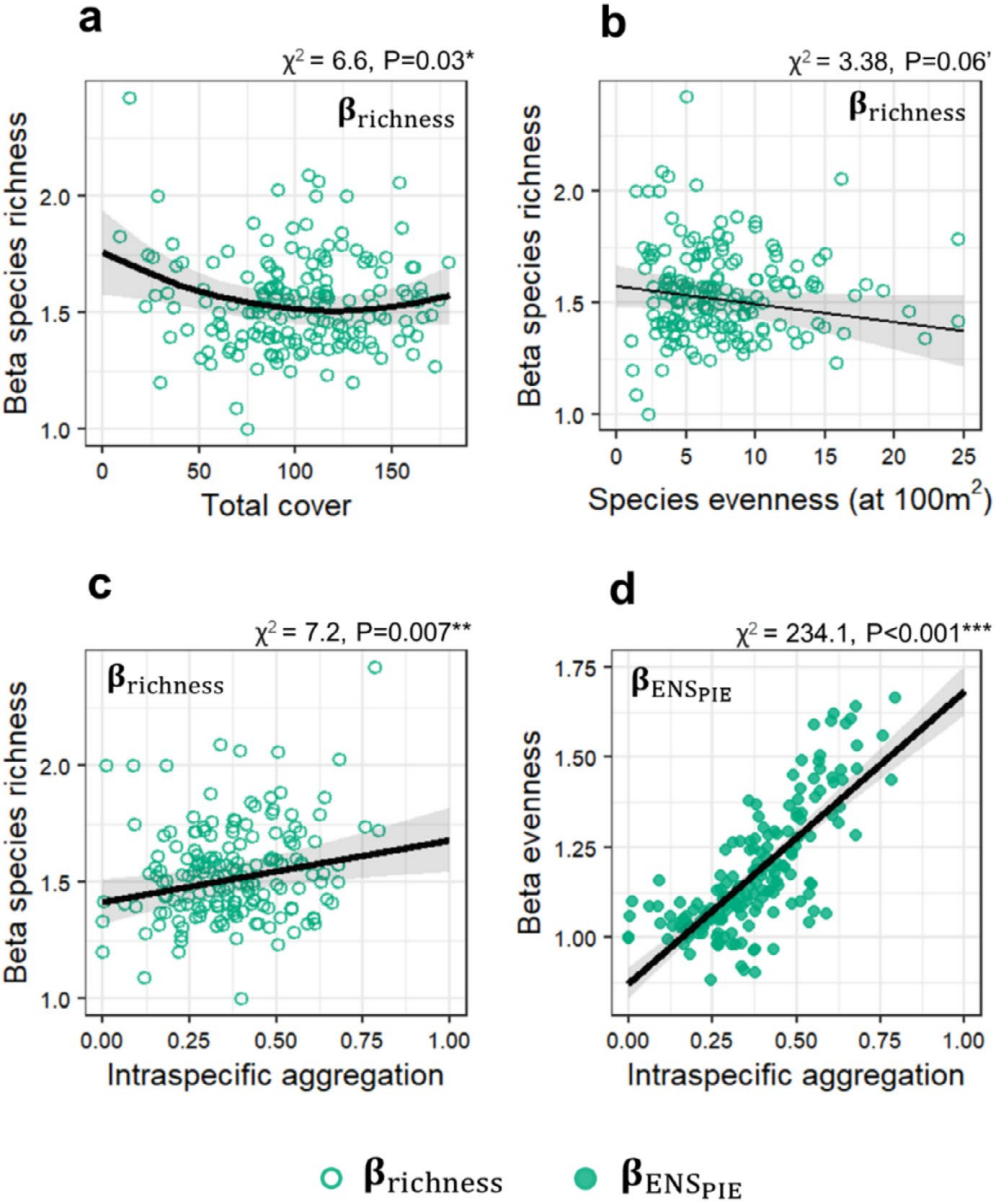
Relationships between **β‐**diversity and proximate factors: total cover, evenness, and intraspecific aggregation.

Most fine‐scale grassland studies have focused on the primacy and the relative importance of biodiversity drivers across grain sizes and extent (Auestad, Rydgren, and Økland 2008; Bergauer et al. 2022; Dembicz et al. 2021b; Kuzemko et al. 2016; Olagoke et al. 2023; Polyakova et al. 2016; Talebi et al. 2021; Turtureanu et al. 2014), while studies investigating the underlying mechanisms of scale dependency remain scarce (e.g., DeMalach et al. 2019). Theoretical models for decoupling these mechanisms (Chase and Knight 2013; Storch, Bohdalková, and Okie 2018) identify species density, evenness, and spatial clustering of conspecifics (intraspecific aggregation) as major mediators of the scale‐dependency of biodiversity drivers. In our study, these mechanisms collectively shaped the scale‐dependency of plant diversity. Specifically, $\beta_{\text{richness}}$ showed a U‐shaped relationship with total cover of plant community (Figure 5a), with scale‐dependency decreasing until mid‐cover levels. At higher cover levels, excessive plant cover reduced species richness at both grain sizes (Figure S4a,b), likely due to asymmetric competition among species, which limited diversity across scales and thereby diminished the scale effects. Additionally, $\beta_{\text{richness}}$ was negatively related to plant community evenness (Figure 5b), likely because higher species evenness allows greater species richness at small scales and thus reduces variability in richness across spatial scales (Chase and Knight 2013). Finally, $\beta_{\text{richness}}$ was positively associated with intraspecific aggregation (Figure 5c), in line with the idea that intraspecific clustering in species spatial distribution reduces richness at small scales by limiting the likelihood of sampling aggregated species, and this effect decreases with increasing sampling area (Chase and Knight 2013; Storch, Bohdalková, and Okie 2018). We also found a strong positive relationship between intraspecific aggregation and $\beta_{{\mathrm{ENS}}_{\mathrm{PIE}}}$ (Figure 5d), which is consistent with theoretical models suggesting that $\beta_{{\mathrm{ENS}}_{\mathrm{PIE}}}$ can reveal whether the scale‐dependency of biodiversity drivers is driven by spatial intraspecific aggregation (Chase and Knight 2013). **β**‐diversity in our study varied across grassland habitat types (Figure 1c), which could suggest an influence of species pool size on the scale‐dependency of biodiversity, as predicted by theory (Chase and Knight 2013). However, disentangling the causal effects of species pool size from the inherent correlations between species richness and species pool size remains a significant challenge in observational studies such as ours (DeMalach et al. 2019; Herben 2000).

**FIGURE 6:**
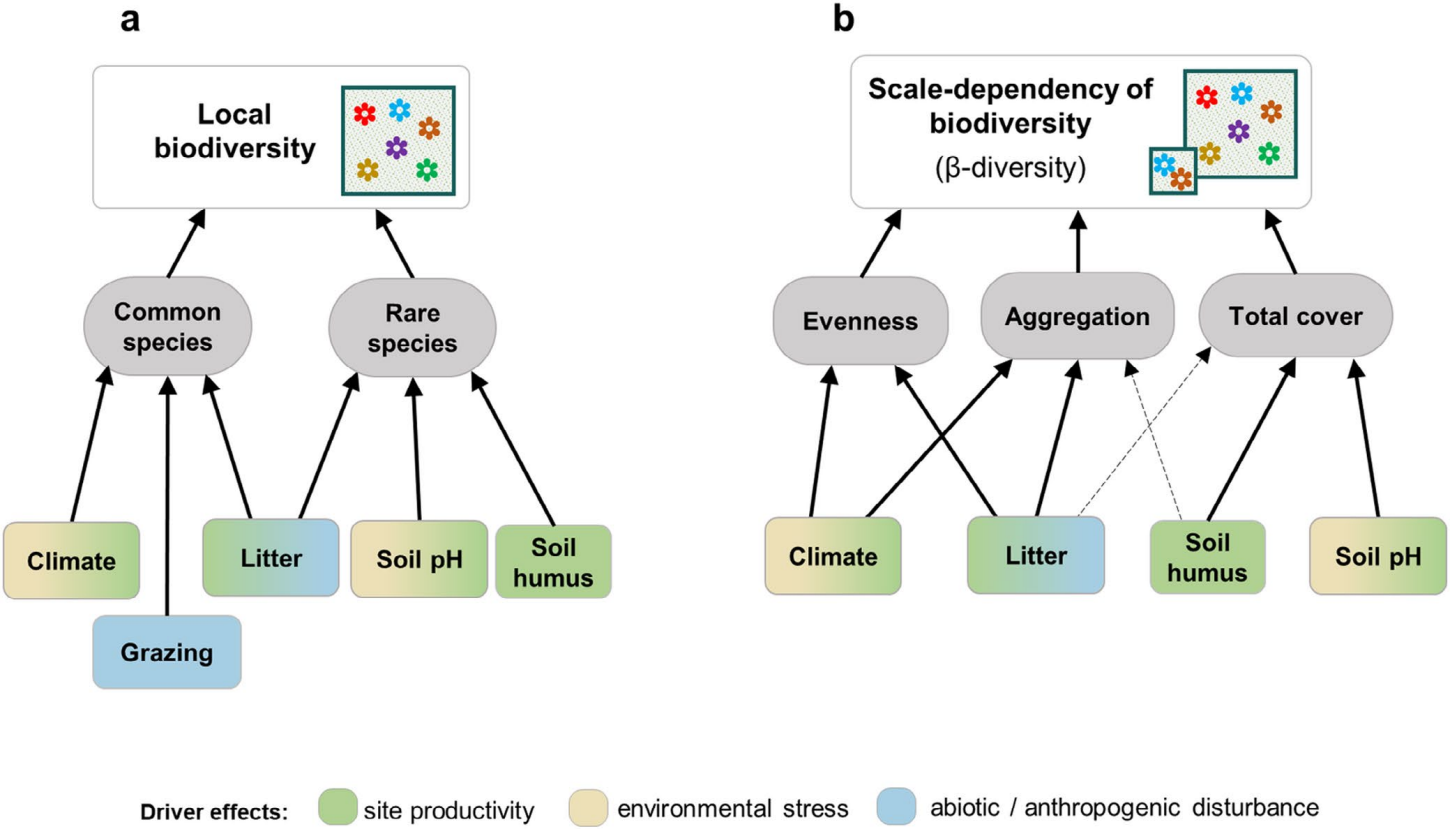
Conceptual diagrams summarizing the results of this study for the mechanisms underlying the effects of the study environmental drivers on local diversity at small (10 m^2^ plots) and larger (100 m^2^ plots) fine‐grain sizes (a) and on **β‐**diversity—the scaling factor among the two scales (b).

We found the hump‐shaped relationship between climate gradient and intraspecific aggregation (Figure S9a). Similarly, we found the hump‐shaped effect of climate gradient on $\beta_{{\mathrm{ENS}}_{\mathrm{PIE}}}$, with a larger difference in community evenness among the two scales toward the middle of the climate gradient (Figure 2d). These results suggest higher intraspecific aggregation in more productive sites (i.e., in a middle of the climate gradient), and align with previous evidence on a positive correlation between productivity proxies and intraspecific aggregation (Chalcraft et al. 2008; Chase and Leibold 2002), which leads to larger effects of productivity on richness at larger spatial scales compared to smaller scales. Our results regarding soil productivity also support this, as we found a strong increase in $\beta_{{\mathrm{ENS}}_{\mathrm{PIE}}}$ at high soil humus levels (Figure 2l) and an increasing trend in species aggregation with higher soil humus content (Figure S9c). Besides aggregation, the scale‐dependency of climate effects on plant diversity operated also via altered species evenness, as we found strong effects of both climate gradient and precipitation variability on plant community evenness (Figure 2b,c). Previous research across a wide gradient of grassland habitat types concluded that the effects of macroclimatic gradients on fine‐grain plant species richness do not depend on grain size (Dembicz et al. 2021a). However, our results suggest that relying on species richness as the sole proxy for biodiversity may underestimate the scale‐dependency of climate and soil effects across different grassland habitat types, due to differences in species pool. Indeed, grassland studies within more climatically uniform regions or habitat types showed more pronounced climate effects on $\beta_{\text{richness}}$, such as in perennial sand grasslands in Hungary (Bartha et al. 2011), Iranian steppes (Talebi et al. 2021), semi‐desert areas in South Africa and Namibia (van der Merwe and van Rooyen 2011), and across global drylands (DeMalach et al. 2019). Unlike species richness, the effects of ecological drivers on ENS_PIE_ are independent of species‐pool size (Chase and Knight 2013), providing a more accurate assessment of the scale‐dependency of biodiversity drivers across diverse grassland habitat types.

Not only the availability of resources (site productivity), but also their patchiness is known to increase the propensity for species to aggregate, which, in turn, may enable species coexistence by reducing interspecific competitive exclusion (Wassmuth et al. 2009) because the competitively weak species generally suffer less from conspecifics than from competitively strong heterospecifics (Stoll and Prati 2001). In our study, litter cover had a hump‐shaped effect on species aggregation (Figure S9e) and on $\beta_{{\mathrm{ENS}}_{\mathrm{PIE}}}$ (Figure 2t), indicating increased intraspecific aggregation toward the middle of the litter‐cover gradient, likely due to a mosaic of microhabitats caused by moderate litter amounts, thus leading to larger effect sizes on community evenness at larger relative to smaller spatial scales (Figure 3a). Furthermore, $\beta_{\text{richness}}$ increased with litter cover (Figure 2s), indicating greater among‐scale difference in species number along the litter gradient. These spatial differences were likely constrained by the limited space of the smaller grain, as the litter effects on richness were strong at 100 m^2^ plots but relatively weak at 10 m^2^ plots (Figure 2q, Figure S10). The space limitation of species occurrences at high disturbance (as caused by excessive litter) are due to higher chances of including more microhabitat patches with increasing area, and thus more species that can occupy these patches (Tamme et al. 2010). The scale‐dependency of litter effects on plant diversity was also mediated by the altered community evenness (litter cover affected evenness, Figure 2r, Figure S5t), but not by total plant cover, as litter had weak effects on cover (Figure S8j). Overall, litter cover was among the key drivers of **β‐**diversity (both$\;\beta_{\text{richness}}$ and $\beta_{{\mathrm{ENS}}_{\mathrm{PIE}}}$, Figure 3b), consistent with previous fine‐scale studies in Romanian dry grasslands (Turtureanu et al. 2014) and Ukrainian steppes (Kuzemko et al. 2016), which showed that the influence of litter cover on plant species richness increases with grain size.

The effect of soil pH on species richness was significantly weaker at smaller than those at larger scale (Figures 3 and 4), leading to a strong U‐shaped effect on $\beta_{\text{richness}}$ (Figure 2o). This indicates that the occurrence of species under soil‐related stress (i.e., toward low and high soil pH) was strongly limited by the area size, which is likely driven by the loss of locally rare species (Schuster and Diekmann 2003). Previous studies in grasslands also found the U‐shaped relationship between soil pH and the fine‐scale **β**‐diversity of plants (Dembicz et al. 2021a). We found no effects of soil pH on $\beta_{{\mathrm{ENS}}_{\mathrm{PIE}}}$ (Figure 2p), intraspecific aggregation (Figure S9d) or on local community evenness (Figure 2n), thus suggesting that the scale‐dependency of soil pH effects were not operating through these mechanisms, but were rather direct, likely due to smaller species pools in more acidic and basic sites. Instead, we found a strong concave‐down decreasing effect of soil pH on total plant cover (Figure S8g,h), indicating that the altered species density could mediate the responses of **β**‐diversity of plants to high levels of soil pH. However, it is important to note the limitations of using plant cover as a proxy for plant density, as plant cover may increase not only with the number of plant individuals but also with their body size (Oksanen 1996). Overall, our results agree with DeMalach et al. (2019), who found that the effects of soil pH on species–area relationship were not mediated by intraspecific aggregation or species evenness in plant communities across global drylands.

Although we found no significant effects of grazing or mowing on **β**‐diversity (Figure 2x), the negative effect of grazing intensity on ENS_PIE_ became more detectable at the larger scale (Figure 2v). These results somewhat align with previous studies suggesting that land use becomes a more important predictor of grassland plant diversity at larger scales (Auestad, Rydgren, and Økland 2008; Spiegelberger et al. 2006; Turtureanu et al. 2014). The scale‐dependency of management effects on plant diversity in grasslands is shown to vary considerably along climatic gradients, e.g. as shown for grazing (de Bello, Lepš, and Sebastià 2007), or management types (Dupré and Diekmann 2001; Spiegelberger et al. 2006). Therefore, the scale‐dependency of land‐use effects may become more detectable in climatically more uniform grassland habitat types, for example as found for the semi‐natural grasslands in Norway (Auestad, Rydgren, and Økland 2008), semi‐natural dry grasslands in Romania (Turtureanu et al. 2014), step grasslands in Ukraine (Kuzemko et al. 2016), and alpine grasslands in Europe (Spiegelberger et al. 2006).

Our study identified key biodiversity drivers and their scale‐dependent effects across different grassland habitat types in Ukraine and defined the mechanisms underlying these effects. Understanding these mechanisms enhances our ability to predict and mitigate the impacts of environmental changes on grassland biodiversity and have important application for management and conservation, as spatial **β**‐diversity is often used to inform biodiversity conservation and management applications (DeMalach et al. 2019; Smith 2010; Socolar et al. 2016; van der Merwe and van Rooyen 2011), and to understand the provisioning of ecosystem functions and services (Mori, Isbell, and Seidl 2018). Our results highlight the need for integrated and adaptive conservation and management strategies tailored to different spatial scales and grassland habitat types. Effective cross‐scale conservation should include prioritizing areas for protection based on biodiversity hotspots identified using different biodiversity facets at different spatial scales, rather than relying solely on local species richness. Monitoring programs should track biodiversity dynamics across spatial scales and explicitly test the underlying mechanisms, enabling the early detection of scale‐specific threats. Our study is observational, and as such, it is subject to the limitations in inferring causal relationships. We call for controlled experimental studies that would explicitly test how species density, evenness, intraspecific aggregation, and species‐pool size mediate and modify the scale‐dependency of biodiversity drivers.

## Author Contributions

**Oksana Buzhdygan:** conceptualization (equal), data curation (equal), formal analysis (lead), funding acquisition (equal), investigation (lead), methodology (lead), project administration (equal), resources (equal), supervision (equal), visualization (equal), writing – original draft (lead), writing – review and editing (lead). **Selina Baldauf:** conceptualization (equal), data curation (equal), formal analysis (equal), methodology (equal), software (lead), visualization (equal), writing – review and editing (equal). **Dariia Borovyk:** conceptualization (supporting), data curation (equal), methodology (equal), visualization (supporting), writing – review and editing (equal). **Denys Vynokurov:** conceptualization (supporting), data curation (equal), methodology (equal), writing – review and editing (equal). **Emma Ladouceur:** conceptualization (equal), formal analysis (supporting), methodology (equal), writing – review and editing (equal). **Olha Chusova:** conceptualization (supporting), data curation (equal), methodology (equal), writing – review and editing (equal). **Svitlana Iemelianova:** conceptualization (supporting), data curation (equal), methodology (equal), writing – review and editing (equal). **Vasyl Budzhak:** conceptualization (supporting), data curation (equal), methodology (equal), writing – review and editing (equal). **Britta Tietjen:** conceptualization (supporting), methodology (equal), writing – review and editing (equal). **Olga Bezrodnova:** data curation (equal), writing – review and editing (supporting). **Olesya Bezsmertna:** data curation (equal), writing – review and editing (supporting). **Illya Chorney:** data curation (equal), writing – review and editing (supporting). **Iwona Dembicz:** data curation (equal), writing – review and editing (supporting). **Jürgen Dengler:** data curation (equal), writing – review and editing (supporting). **Yakiv Didukh:** data curation (equal), writing – review and editing (supporting). **Monika Janišová:** data curation (equal), writing – review and editing (supporting). **Oleksandr Khodosovtsev:** data curation (equal), writing – review and editing (supporting). **Oksana Kucher:** data curation (equal), writing – review and editing (supporting). **Ivan Moysiyenko:** data curation (equal), writing – review and editing (supporting). **Alla Tokariuk:** data curation (equal), writing – review and editing (supporting). **Iuliia Vasheniak:** data curation (equal), writing – review and editing (supporting). **Olena Yavorska:** data curation (equal), writing – review and editing (supporting). **Jonathan Chase:** conceptualization (equal), formal analysis (supporting), funding acquisition (supporting), methodology (equal), resources (supporting), supervision (supporting), writing – review and editing (equal). **Anna Kuzemko:** conceptualization (equal), data curation (equal), funding acquisition (equal), investigation (equal), methodology (equal), project administration (equal), resources (equal), supervision (equal), writing – review and editing (equal).

## Conflicts of Interest

The authors declare no conflicts of interest.

## Code Availability

The codes for the analyses of this study are available at https://doi.org/10.5281/zenodo.14803138.

## Supporting information


Data S1.

## Data Availability

All data used to support the conclusions of this study are available at https://doi.org/10.5281/zenodo.14803138.

## References

[ece370941-bib-0001] Auestad, I. , K. Rydgren , and R. H. Økland . 2008. “Scale‐Dependence of Vegetation‐Environment Relationships in Semi‐Natural Grasslands.” Journal of Vegetation Science 19, no. 1: 139–148. 10.3170/2007-8-18344.

[ece370941-bib-0002] Bartha, S. , G. Campetella , M. Kertész , et al. 2011. “Beta Diversity and Community Differentiation in Dry Perennial Sand Grasslands.” Annali Di Botanica 1: 9–18.

[ece370941-bib-0003] Barton, P. S. , S. A. Cunningham , A. D. Manning , H. Gibb , D. B. Lindenmayer , and R. K. Didham . 2013. “The Spatial Scaling of Beta Diversity.” Global Ecology and Biogeography 22, no. 6: 639–647. 10.1111/geb.12031.

[ece370941-bib-0004] Baselga, A. 2017. “Partitioning Abundance‐Based Multiple‐Site Dissimilarity Into Components: Balanced Variation in Abundance and Abundance Gradients.” Methods in Ecology and Evolution 8, no. 7: 799–808. 10.1111/2041-210X.12693.

[ece370941-bib-0005] Baselga, A. , and C. D. L. Orme . 2012. “Betapart: An R Package for the Study of Beta Diversity.” Methods in Ecology and Evolution 3, no. 5: 808–812. 10.1111/j.2041-210X.2012.00224.x.

[ece370941-bib-0006] Bates, D. , M. Maechler , B. Bolker , and S. Walker . 2015. “Fitting Linear Mixed‐Effects Models Using lme4.” Journal of Statistical Software 67, no. 1: 1–48. 10.18637/jss.v067.i01.

[ece370941-bib-0007] Bergauer, M. , I. Dembicz , S. Boch , et al. 2022. “Scale‐Dependent Patterns and Drivers of Vascular Plant, Bryophyte and Lichen Diversity in Dry Grasslands of the Swiss Inneralpine Valleys.” Alpine Botany 132, no. 2: 195–209. 10.1007/s00035-022-00285-y.

[ece370941-bib-0008] Biurrun, I. , R. Pielech , I. Dembicz , and F. Gillet . 2021. “Benchmarking Plant Diversity of Palaearctic Grasslands and Other Open Habitats.” Journal of Vegetation Science 32: e13050. 10.1111/jvs.13050.

[ece370941-bib-0009] Blowes, S. A. , G. N. Daskalova , M. Dornelas , et al. 2022. “Local Biodiversity Change Reflects Interactions Among Changing Abundance, Evenness, and Richness.” Ecology 103, no. 12: e3820. 10.1002/ecy.3820. 35869831

[ece370941-bib-0010] Borovyk, D. , I. Dembicz , J. Dengler , et al. 2023. “Plant Species Richness Records in Ukrainian Steppes.” Tuexenia 44: 225–239. 10.14471/2024.44.002.

[ece370941-bib-0011] Buzhdygan, O. Y. , S. T. Meyer , W. W. Weisser , et al. 2020a. “Biodiversity Increases Multitrophic Energy Use Efficiency, Flow and Storage in Grasslands.” Nature Ecology & Evolution 4, no. 3: 393–405. 10.1038/s41559-020-1123-8. 32094542

[ece370941-bib-0012] Buzhdygan, O. Y. , B. Tietjen , S. S. Rudenko , V. A. Nikorych , and J. S. Petermann . 2020b. “Direct and Indirect Effects of Land‐Use Intensity on Plant Communities Across Elevation in Semi‐Natural Grasslands.” PLoS One 15, no. 11: e0231122. 10.1371/journal.pone.0231122. 33232338 PMC7685434

[ece370941-bib-0013] Chalcraft, D. R. , S. B. Cox , C. Clark , et al. 2008. “Scale‐Dependent Responses of Plant Biodiversity to Nitrogen Enrichment.” Ecology 89, no. 8: 2165–2171. 10.1890/07-0971.1. 18724726

[ece370941-bib-0014] Chao, A. , C. H. Chiu , and L. Jost . 2014. “Unifying Species Diversity, Phylogenetic Diversity, Functional Diversity, and Related Similarity and Differentiation Measures Through Hill Numbers.” Annual Review of Ecology, Evolution, and Systematics 45: 297–324. 10.1146/annurev-ecolsys-120213-091540.

[ece370941-bib-0015] Chase, J. M. , and T. M. Knight . 2013. “Scale‐Dependent Effect Sizes of Ecological Drivers on Biodiversity: Why Standardised Sampling Is Not Enough.” Ecology Letters 16, no. Suppl 1: 17–26. 10.1111/ele.12112. 23679009

[ece370941-bib-0016] Chase, J. M. , and M. A. Leibold . 2002. “Spatial Scale Dictates the Productivity‐Biodiversity Relationship.” Nature 416: 427–430. 11919631 10.1038/416427a

[ece370941-bib-0017] Chase, J. M. , B. J. McGill , D. J. McGlinn , et al. 2018. “Embracing Scale‐Dependence to Achieve a Deeper Understanding of Biodiversity and Its Change Across Communities.” Ecology Letters 21, no. 11: 1737–1751. 10.1111/ele.13151. 30182500

[ece370941-bib-0018] Chase, J. M. , B. J. Mcgill , P. L. Thompson , et al. 2019. “Species Richness Change Across Spatial Scales.” Oikos 128: 1079–1091. 10.1111/oik.05968.

[ece370941-bib-0019] Chytrý, M. , A. Chiarucci , M. Pärtel , et al. 2019. “Progress in Vegetation Science: Trends Over the Past Three Decades and New Horizons.” Journal of Vegetation Science 30, no. 1: 1–4. 10.1111/jvs.12697.

[ece370941-bib-0020] Chytrý, M. , J. Danihelka , N. Ermakov , et al. 2007. “Plant Species Richness in Continental Southern Siberia: Effects of pH and Climate in the Context of the Species Pool Hypothesis.” Global Ecology and Biogeography 16, no. 5: 668–678. 10.1111/j.1466-8238.2007.00320.x.

[ece370941-bib-0021] Chytrý, M. , T. Dražil , M. Hájek , et al. 2015. “The Most Species‐Rich Plant Communities in The Czech Republic and Slovakia (With New World Records).” Preslia 87, no. 3: 217–278.

[ece370941-bib-0022] Chytrý, M. , and Z. Otýpková . 2003. “Plot Sizes Used for Phytosociological Sampling of European Vegetation.” Journal of Vegetation Science 14, no. 4: 563–570. 10.1111/j.1654-1103.2003.tb02183.x.

[ece370941-bib-0023] Chytrý, M. , L. Tichý , S. M. Hennekens , et al. 2020. “EUNIS Habitat Classification: Expert System, Characteristic Species Combinations and Distribution Maps of European Habitats.” Applied Vegetation Science 23, no. 4: 648–675. 10.1111/avsc.12519.

[ece370941-bib-0024] Chytrý, M. , L. Tichý , and J. Rolecek . 2003. “Local and Regional Patterns of Species Richness Ph/Calclum Gradient.” Folia Geobotanica 38: 429–442.

[ece370941-bib-0025] de Bello, F. , J. Lepš , and M.‐T. Sebastià . 2007. “Grazing Effects on the Species‐Area Relationship: Variation Along a Climatic Gradient in NE Spain.” Journal of Vegetation Science 18, no. 1: 25. 10.1658/1100-9233(2007)18[25:geotsr]2.0.co;2.

[ece370941-bib-0026] DeMalach, N. , H. Saiz , E. Zaady , and F. T. Maestre . 2019. “Plant Species–Area Relationships Are Determined by Evenness, Cover and Aggregation in Drylands Worldwide.” Global Ecology and Biogeography 28, no. 3: 290–299. 10.1111/geb.12849. 30886537 PMC6420124

[ece370941-bib-0027] Dembicz, I. , J. Dengler , M. J. Steinbauer , et al. 2021a. “Fine‐Grain Beta Diversity of Palaearctic Grassland Vegetation.” Journal of Vegetation Science 32: e13045. 10.1111/jvs.13045.

[ece370941-bib-0028] Dembicz, I. , N. Velev , S. Boch , et al. 2021b. “Drivers of Plant Diversity in Bulgarian Dry Grasslands Vary Across Spatial Scales and Functional‐Taxonomic Groups.” Journal of Vegetation Science 32, no. 1: e12935. 10.1111/jvs.12935.

[ece370941-bib-0029] Dengler, J. , I. Biurrun , S. Boch , I. Dembicz , and P. Torok . 2020. “Grasslands of the Palaearctic Biogeographic Realm: Introduction and Synthesis.” In Encyclopedia of the World's Biomes (Vols. 3–5, Issue iDiv). Amsterdam, Netherlands: Elsevier. 10.1016/B978-0-12-409548-9.12432-7.

[ece370941-bib-0030] Dengler, J. , S. Boch , G. Filibeck , et al. 2016. “Assessing Plant Diversity and Composition in Grass ‐ Lands Across Spatial Scales: The Standardised EDGG Sampling Methodology.” Bulletin of the Eurasian Dry Grassland Group 32: 13–30.

[ece370941-bib-0031] Dengler, J. , and I. Dembicz . 2023. “Should We Estimate Plant Cover in Percent or on Ordinal Scales?” Vegetation Classification and Survey 4: 131–138. 10.3897/VCS.98379.

[ece370941-bib-0032] Díaz, S. , and Y. Malhi . 2022. “Biodiversity: Concepts, Patterns, Trends, and Perspectives.” Annual Review of Environment and Resources 47: 31–63.

[ece370941-bib-0033] Díaz, S. , J. Settele , E. S. Brondízio , et al. 2019. “Pervasive Human‐Driven Decline of Life on Earth Points to the Need for Transformative Change.” Science 366, no. 6471: eaax3100. 10.1126/science.aax3100. 31831642

[ece370941-bib-0034] Dupré, C. , and M. Diekmann . 2001. “Differences in Species Richness and Life‐History Traits Between Grazed and Abandoned Grasslands in Southern Sweden.” Ecography 24, no. 3: 275–286. 10.1111/j.1600-0587.2001.tb00200.x.

[ece370941-bib-0035] Enyedi, Z. M. , E. Ruprecht , and M. Deák . 2008. “Long‐Term Effects of the Abandonment of Grazing on Steppe‐Like Grasslands.” Applied Vegetation Science 11, no. 1: 55–62. 10.3170/2007-7-18316.

[ece370941-bib-0036] Euro+Med . “2006+ [Continuously Updated]: Euro+Med PlantBase ‐ The Information Resource for Euro‐Mediterranean Plant Diversity.” 2023. http://www.europlusmed.org.

[ece370941-bib-0037] Evans, D. 2016. “Habitat Complexes, a Neglected Part of the Eunis Habitats Classification?” *25th Meeting of European Vegetation Survey Roma (Italy)*, 35.

[ece370941-bib-0038] Facelli, J. M. , and S. T. A. Pickett . 1991. “Plant Litter: Its Dynamics and Effects on Plant Community Structure.” Botanical Review 57, no. 1: 1–32. 10.1007/bf02858763.

[ece370941-bib-0039] Field, R. , B. A. Hawkins , H. V. Cornell , et al. 2009. “Spatial Species‐Richness Gradients Across Scales: A Meta‐Analysis.” Journal of Biogeography 36, no. 1: 132–147. 10.1111/j.1365-2699.2008.01963.x.

[ece370941-bib-0040] Filibeck, G. , M. G. Sperandii , M. Bazzichetto , L. D. Mancini , F. Rossini , and L. Cancellieri . 2019. “Exploring the Drivers of Vascular Plant Richness at Very Fine Spatial Scale in Sub‐Mediterranean Limestone Grasslands (Central Apennines, Italy).” Biodiversity and Conservation 28, no. 10: 2701–2725. 10.1007/s10531-019-01788-7.

[ece370941-bib-0041] Gaston, K. J. 2000. “Global Patterns in Biodiversity.” Nature 405: 220–227. 10821282 10.1038/35012228

[ece370941-bib-0042] Grime, J. P. 1979. Plant Strategies, Vegetation Processes, and Ecosystem Properties. Hoboken, NJ: John Wiley & Sons, Ltd.

[ece370941-bib-0043] Hartig, F. 2022. “DHARMa: Residual Diagnostics for Hierarchical (Multi‐Level/Mixed) Regression Models.” R Package. https://cran.r‐project.org/package=DHARM.

[ece370941-bib-0044] He, F. , and P. Legendre . 2002. “Species Diversity Patterns Derived From Species–Area Models.” Ecology 83: 1185–1198. 10.1890/0012-9658(2002)083[1185:SDPDFS]2.0.CO;2.

[ece370941-bib-0045] Herben, T. 2000. “Correlation Between Richness per Unit Area and the Species Pool Cannot Be Used to Demonstrate the Species Pool Effect.” Journal of Vegetation Science 11, no. 1: 123–126. 10.2307/3236783.

[ece370941-bib-0046] Hillebrand, H. , B. Blasius , E. T. Borer , et al. 2018. “Biodiversity Change Is Uncoupled From Species Richness Trends: Consequences for Conservation and Monitoring.” Journal of Applied Ecology 55, no. 1: 169–184. 10.1111/1365-2664.12959.

[ece370941-bib-0047] Hodgetts, N. G. , L. Söderström , T. L. Blockeel , et al. 2020. “An Annotated Checklist of Bryophytes of Europe, Macaronesia and Cyprus.” Journal of Bryology 42, no. 1: 1–116. 10.1080/03736687.2019.1694329.

[ece370941-bib-0048] Jaeger, B. 2017. “r2glmm: Computes R Squared for Mixed (Multilevel) Models.” R Package Version 0.1.2 (0.1.2). https://github.com/bcjaeger/r2glmm.

[ece370941-bib-0049] Jost, L. 2006. “Entropy and Diversity.” Oikos 113, no. 2: 363–375. 10.1111/j.2006.0030-1299.14714.x.

[ece370941-bib-0050] Karger, D. N. , O. Conrad , J. Böhner , et al. 2018. “Data From: Climatologies at High Resolution for the Earth's Land Surface Areas.” Scientific Data 4: 170122. 10.16904/envidat.228.v2.1. PMC5584396 28872642

[ece370941-bib-0051] Kondratyuk, S. Y. , L. P. Popova , O. Y. Khodosovtsev , L. Lokös , N. M. Fedorenko , and N. V. Kapets . 2021. “The Fourth Checklist of Ukrainian Lichen‐Forming and Lichenicolous Fungi With Analysis of Current Additions.” Acta Botanica Hungarica 63, no. 1–2: 97–163. 10.1556/034.63.2021.1-2.8.

[ece370941-bib-0052] Kuzemko, A. , V. Budzhak , Y. Vasheniak , et al. 2022. “Atlas of Grassland Habitats of Ukraine.” [In Ukrainian]. Druk Art.

[ece370941-bib-0053] Kuzemko, A. , M. J. Steinbauer , T. Becker , et al. 2016. “Patterns and Drivers of Phytodiversity in Steppe Grasslands of Central Podolia (Ukraine).” Biodiversity and Conservation 25, no. 12: 2233–2250. 10.1007/s10531-016-1060-7.

[ece370941-bib-0054] Ladouceur, E. , F. Isbell , A. T. Clark , et al. 2023. “The Recovery of Plant Community Composition Following Passive Restoration Across Spatial Scales, December 2022, 1–16.” Journal of Ecology 111, no. 4: 814–829. 10.1111/1365-2745.14063.

[ece370941-bib-0055] Lamb, E. G. 2008. “Direct and Indirect Control of Grassland Community Structure by Litter, Resources, and Biomass.” Ecology 89, no. 1: 216–225. 10.1890/07-0393.1. 18376563

[ece370941-bib-0056] May, F. , K. Gerstner , D. J. McGlinn , X. Xiao , and J. M. Chase . 2018. “Mobsim: An r Package for the Simulation and Measurement of Biodiversity Across Spatial Scales.” Methods in Ecology and Evolution 9, no. 6: 1401–1408. 10.1111/2041-210X.12986.

[ece370941-bib-0057] McGill, B. J. 2010a. “Matters of Scale.” Science 328, no. 5978: 575–576. 10.1126/science.1188528. 20431001

[ece370941-bib-0058] McGill, B. J. 2010b. “Towards a Unification of Unified Theories of Biodiversity.” Ecology Letters 13, no. 5: 627–642. 10.1111/j.1461-0248.2010.01449.x. 20337695

[ece370941-bib-0059] McGill, B. J. 2011. “Linking Biodiversity Patterns by Autocorrelated Random Sampling.” American Journal of Botany 98, no. 3: 481–502. 10.3732/ajb.1000509. 21613141

[ece370941-bib-0060] Michalet, R. , R. W. Brooker , L. A. Cavieres , et al. 2006. “Do Biotic Interactions Shape Both Sides of the Humped‐Back Model of Species Richness in Plant Communities?” Ecology Letters 9, no. 7: 767–773. 10.1111/j.1461-0248.2006.00935.x. 16796565

[ece370941-bib-0061] Mori, A. S. , F. Isbell , and R. Seidl . 2018. “β‐Diversity, Community Assembly, and Ecosystem Functioning.” Trends in Ecology & Evolution 33, no. 7: 549–564. 10.1016/j.tree.2018.04.012. 29807839 PMC7612777

[ece370941-bib-0062] Moysiyenko, I. , D. Vynokurov , D. Shyriaieva , et al. 2022. “Grasslands and Coastal Habitats of Southern Ukraine: First Results From the 15th EDGG Field Workshop.” Palaearctic Grasslands ‐ Journal of the Eurasian Dry Grassland Group 2022, no. 52: 44–83. 10.21570/edgg.pg.52.44-83.

[ece370941-bib-0063] Newbold, T. , L. N. Hudson , S. L. L. Hill , et al. 2015. “Global Effects of Land Use on Local Terrestrial Biodiversity.” Nature 520, no. 7545: 45–50. 10.1038/nature14324. 25832402

[ece370941-bib-0064] Oksanen, A. J. , F. G. Blanchet , R. Kindt , et al. 2018. “Vegan: Community Ecology Package.” 10.4135/9781412971874.n145.

[ece370941-bib-0065] Oksanen, J. 1996. “Is the Humped Relationship Between Species Richness and Biomass an Artefact due to Plot Size?” Journal of Ecology 84, no. 2: 293. 10.2307/2261364.

[ece370941-bib-0066] Olagoke, A. , F. Jeltsch , B. Tietjen , U. Berger , H. Ritter , and S. Maaß . 2023. “Small‐Scale Heterogeneity Shapes Grassland Diversity in Low‐To‐Intermediate Resource Environments.” Journal of Vegetation Science 34, no. 4: e13196. 10.1111/jvs.13196.

[ece370941-bib-0067] Petermann, J. S. , and O. Y. Buzhdygan . 2021. “Grassland Biodiversity.” Current Biology 31, no. 19: R1195–R1201. 10.1016/j.cub.2021.06.060. 34637731

[ece370941-bib-0068] Polyakova, M. A. , I. Dembicz , T. Becker , et al. 2016. “Scale‐ and Taxon‐Dependent Patterns of Plant Diversity in Steppes of Khakassia, South Siberia (Russia).” Biodiversity and Conservation 25, no. 12: 2251–2273. 10.1007/s10531-016-1093-y.

[ece370941-bib-0069] Primack, R. B. , A. J. Miller‐Rushing , R. T. Corlett , et al. 2018. “Biodiversity Gains? The Debate on Changes in Local‐Vs Global‐Scale Species Richness.” Biological Conservation 219: A1–A3.

[ece370941-bib-0070] R Core Team . 2022. R: A Language and Environment for Statistical Computing. Vienna, Austria: R Foundation for Statistical Computing. https://www.r‐project.org.

[ece370941-bib-0071] Rajaniemi, T. K. 2003. “Explaining Productivity‐Diversity Relationships in Plants.” Oikos 101, no. 3: 449–457. 10.1034/j.1600-0706.2003.12128.x.

[ece370941-bib-0072] Roleček, J. , P. Dřevojan , P. Hájková , and M. Hájek . 2019. “Report of New Maxima of Fine‐Scale Vascular Plant Species Richness Recorded in East‐Central European Semi‐Dry Grasslands.” Tuexenia 39, no. March: 423–431. 10.14471/2019.39.008.

[ece370941-bib-0073] Roswell, M. , J. Dushoff , and R. Winfree . 2021. “A Conceptual Guide to Measuring Species Diversity.” Oikos 130, no. 3: 321–338. 10.1111/oik.07202.

[ece370941-bib-0074] Ruprecht, E. , M. Z. Enyedi , R. L. Eckstein , and T. W. Donath . 2010. “Restorative Removal of Plant Litter and Vegetation 40 Years After Abandonment Enhances Re‐Emergence of Steppe Grassland Vegetation.” Biological Conservation 143, no. 2: 449–456. 10.1016/j.biocon.2009.11.012.

[ece370941-bib-0075] Ruprecht, E. , and A. Szabó . 2012. “Grass Litter Is a Natural Seed Trap in Long‐Term Undisturbed Grassland.” Journal of Vegetation Science 23, no. 3: 495–504. 10.1111/j.1654-1103.2011.01376.x.

[ece370941-bib-0076] Sala, O. E. , F. S. Chapin III , J. J. Armesto , et al. 2000. “Global Biodiversity Scenarios for the Year 2100.” Science (New York, N.Y.) 287: 1770–1774. 10.1126/science.287.5459.1770. 10710299

[ece370941-bib-0077] Schaminée, J. H. J. , S. M. Hennekens , J. A. M. Janssen , and J. S. Rodwell . 2018. “Updated Crosswalk of the Revised EUNIS Habitat Classification With the European Vegetation Classification and Indicator Species for the EUNIS Grassland, Shrubland and Forest Types.”

[ece370941-bib-0078] Schuster, B. , and M. Diekmann . 2003. “Changes in Species Density Along the Soil pH Gradient — Evidence From German Plant Communities.” Folia Geobotanica 38: 367–379.

[ece370941-bib-0079] Shapoval, V. , and A. Kuzemko . 2021. “Syntaxonomy of Steppe Depression Vegetation of Ukraine.” Vegetation Classification and Survey 2: 87–108. 10.3897/VCS/2021/62825.

[ece370941-bib-0080] Siefert, A. , C. Ravenscroft , D. Althoff , et al. 2012. “Scale Dependence of Vegetation‐Environment Relationships: A Meta‐Analysis of Multivariate Data.” Journal of Vegetation Science 23, no. 5: 942–951. 10.1111/j.1654-1103.2012.01401.x.

[ece370941-bib-0081] Šímová, I. , Y. M. Li , and D. Storch . 2013. “Relationship Between Species Richness and Productivity in Plants: The Role of Sampling Effect, Heterogeneity and Species Pool.” Journal of Ecology 101, no. 1: 161–170. 10.1111/1365-2745.12011.

[ece370941-bib-0082] Smith, A. B. 2010. “Caution With Curves: Caveats for Using the Species‐Area Relationship in Conservation.” Biological Conservation 143, no. 3: 555–564. 10.1016/j.biocon.2009.11.003.

[ece370941-bib-0083] Socolar, J. B. , J. J. Gilroy , W. E. Kunin , and D. P. Edwards . 2016. “How Should Beta‐Diversity Inform Biodiversity Conservation? Conservation Targets at Multiple Spatial Scales.” Trends in Ecology & Evolution 31: 67–80. https://ac.els‐cdn.com/S016953471500289X/1‐s2.0‐S016953471500289X‐main.pdf?_tid=0aee4430‐0bd4‐11e8‐a91a‐00000aacb35e&acdnat=1517986778_0612927d48bf168559f8e9159f109ba4. 26701706 10.1016/j.tree.2015.11.005

[ece370941-bib-0084] Spiegelberger, T. , D. Matthies , H. Müller‐Schärer , and U. Schaffner . 2006. “Scale‐Dependent Effects of Land Use on Plant Species Richness of Mountain Grassland in the European Alps.” Ecography 29, no. 4: 541–548. 10.1111/j.0906-7590.2006.04631.x.

[ece370941-bib-0085] Srivastava, D. S. S. , and J. H. Lawton . 1998. “Why More Productive Sites Have More Species: An Experimental Test of Theory Using Tree Hole Communities.” American Naturalist 152, no. 4: 510–529. 10.1086/286187. 18811361

[ece370941-bib-0086] Stein, A. , K. Gerstner , and H. Kreft . 2014. “Environmental Heterogeneity as a Universal Driver of Species Richness Across Taxa, Biomes and Spatial Scales.” Ecology Letters 17, no. 7: 866–880. 10.1111/ele.12277. 24751205

[ece370941-bib-0087] Stoll, P. , and D. Prati . 2001. “Intraspecific Aggregation Alters Competitive Interactions in Experimental Plant Communities.” Ecology 82, no. 2: 319–327. 10.1890/0012-9658(2001)082[0319:IAACII]2.0.CO;2.

[ece370941-bib-0088] Storch, D. 2016. “The Theory of the Nested Species–Area Relationship: Geometric Foundations of Biodiversity Scaling.” Journal of Vegetation Science 27, no. 5: 880–891. 10.1111/jvs.12428.

[ece370941-bib-0089] Storch, D. , E. Bohdalková , and J. Okie . 2018. “The More‐Individuals Hypothesis Revisited: The Role of Community Abundance in Species Richness Regulation and the Productivity–Diversity Relationship.” Ecology Letters 21, no. 6: 920–937. 10.1111/ele.12941. 29659144

[ece370941-bib-0090] Talebi, A. , F. Attar , A. Naqinezhad , I. Dembicz , and J. Dengler . 2021. “Scale‐Dependent Patterns and Drivers of Plant Diversity in Steppe Grasslands of the Central Alborz Mts., Iran.” Journal of Vegetation Science 32, no. 2: 1–15. 10.1111/jvs.13005.

[ece370941-bib-0091] Tamme, R. , I. Hiiesalu , L. Laanisto , R. Szava‐Kovats , and M. Pärtel . 2010. “Environmental Heterogeneity, Species Diversity and Co‐Existence at Different Spatial Scales.” Journal of Vegetation Science 21, no. 4: 796–801. 10.1111/j.1654-1103.2010.01185.x.

[ece370941-bib-0092] Tjørve, E. , W. E. Kunin , C. Polce , and K. M. Calf Tjørve . 2008. “Species‐Area Relationship: Separating the Effects of Species Abundance and Spatial Distribution.” Journal of Ecology 96, no. 6: 1141–1151. 10.1111/j.1365-2745.2008.01433.x.

[ece370941-bib-0093] Török, P. , M. Janišová , A. Kuzemko , S. Rusina , and Z. D. Stevanovic . 2018. “Grasslands, Their Threats and Management in Eastern Europe.” In Grasslands of the World: Diversity, Management and Conservation, edited by V. R. Squires , J. Dengler , L. Hua , and H. Feng , 64–88. Boca Raton, FL: CRC Press, Taylor & Francis Group. 10.1201/9781315156125.

[ece370941-bib-0094] Turtureanu, P. D. , S. Palpurina , T. Becker , et al. 2014. “Scale‐ and Taxon‐Dependent Biodiversity Patterns of Dry Grassland Vegetation in Transylvania.” Agriculture, Ecosystems and Environment 182: 15–24. 10.1016/j.agee.2013.10.028.

[ece370941-bib-0095] Ulrich, W. , S. Soliveres , F. T. Maestre , et al. 2014. “Climate and Soil Attributes Determine Plant Species Turnover in Global Drylands.” Journal of Biogeography 41, no. 12: 2307–2319. 10.1111/jbi.12377. 25914437 PMC4407967

[ece370941-bib-0096] van der Merwe, H. , and M. W. van Rooyen . 2011. “Species‐Area Relationships in the Hantam‐Tanqua‐Roggeveld, Succulent Karoo, South Africa.” Biodiversity and Conservation 20, no. 6: 1183–1201. 10.1007/s10531-011-0022-3.

[ece370941-bib-0097] Wassmuth, B. E. , P. Stoll , T. Tscharntke , and C. Thies . 2009. “Spatial Aggregation Facilitates Coexistence and Diversity of Wild Plant Species in Field Margins.” Perspectives in Plant Ecology, Evolution and Systematics 11, no. 2: 127–135. 10.1016/j.ppees.2009.02.001.

[ece370941-bib-0098] Whittaker, R. H. 1972. “Evolution and Measurement of Species Diversity.” Taxon 21, no. 2/3: 213–251.

[ece370941-bib-0099] Wilson, J. B. , R. K. Peet , J. Dengler , and M. Pärtel . 2012. “Plant Species Richness: The World Records.” Journal of Vegetation Science 23, no. 4: 796–802. 10.1111/j.1654-1103.2012.01400.x.

