# Supplementary material for: Scale‐Dependent Effects of Plant Diversity Drivers Across Different Grassland Habitats in Ukraine
Source: Ecol Evol. 2025 Feb 12;15(2):e70941. doi: 10.1002/ece3.70941 (PMC11815339; doi:10.1002/ece3.70941)
Supplement: Supplementary file 1 — Data S1. [file ECE3-15-e70941-s001.docx]

**Section 1. Supplementary methods**

***1.1 Calculation of standardized effects***

We evaluated the relative importance of the study predictors as drivers of plant diversity at each sampling scale by comparing their standardized effect sizes (Fig. 3a). For this, for each predictor ***x*** with a unimodal effect in the final (G)LMM model, we created the composite variable arising from the combined effects of both unsquared and squared terms of that predictor (***x*** and ***x***^2^). For this, the parameter estimates of ***x*** and ***x***^2^ were used as the loadings (weights) for the composite variable, where ***x*** and ***x***^2^ are multiplied by their loading and summed to generate the factor scores for the composite variable: b_1_×***x*** + b_2_×***x***^2^. The final (G)LMM was then refitted with the unimodal effect replaced with the composite variable. The parameter estimate of this composite variable serves as the combined effect of both the linear and non-linear effects of the target predictor ***x***. For details on the methods of using statistical composites for summarizing the collective nonlinear effects see (Grace & Bollen, 2008; J. Lefcheck, 2021). To be able to interpret and compare the parameter estimates on a comparable scale we have standardized the obtained estimate coefficients using the ‘*coefs*’ function from the ‘*piecewiseSEM*’ package in R (J. S. Lefcheck, 2016). In addition to the standardized effects, we also compared the relative variance explained by each driver on each sampling scale (Fig. 3c) by calculating partial-R^2^ from the (G)LMMs using the ‘*r2beta*’ function from the ‘*r2glmm*’ package in R (Jaeger, 2017).

**Section 2. Supplementary discussion: Effects of soil properties and litter cover on local plant diversity**

***2.1 Effects of soil properties***

We found hump-shaped effects of both soil humus content and soil pH on species richness at both spatial scales (Fig. 2i, Fig. 2m), with soil humus showing relatively stronger effects (Fig. 3). Our results are consistent with previous studies that found soil organic carbon as a strong driver of species richness at fine spatial scales (Polyakova et al., 2016; Turtureanu et al., 2014). The hump-shaped effects of soil humus on species richness may be attributed to site productivity effects, as in most cases the soil humus content in grasslands is a measure of soil fertility and water retention, and thus can be considered as proxy for aboveground productivity. According to the *more individuals hypothesis*, higher resource availability allows species to maintain larger populations in a given area, buffering these species against extinction, and thus leading to higher species richness in more productive sites (Srivastava & Lawton, 1998). However, as plant cover increases with soil fertility (Fig. S8e-f), i.e. due to the larger population sizes and/or larger body size of individuals, the asymmetric competition among species due to space limitation causes species loss (Storch et al., 2018). In our study, the relationship between species richness and cover followed a hump-shaped pattern, thus supporting the *more individuals hypothesis* until the point where the high plant cover led to a reduction in species number (Fig. S4a-b), suggesting the asymmetric competition at the high end of the plant cover gradient.

The hump-shaped effect of soil pH on species richness can be related to the stress gradient, where the uptake of nutrients by plants is limited at low and high levels of soil pH and with toxicity at low soil pH due to exchangeable aluminium in acidic soils (Chytrý et al., 2007; Tyler, 2003). It is important to note that the highest pH values in our study were found in saline grasslands and in the dry and hot grassland habitats containing the driest soils with pronounced summer droughts and winter frosts (Fig. S2a). Despite statistical adjustments for the climate gradient in assessing soil pH effects (see “Materials and methods” in the main text), it remains challenging to disentangle the physiological effects of climatic stress (Chytrý et al., 2007; Palpurina et al., 2017) and soil salinity (Deák et al., 2014; Polyakova et al., 2016) using observational data.

Evolutionary species-pool effects could also contribute to the observed in our study low species richness at the high end of the soil humus gradient and at low soil pH levels (Fig. 2i, Fig. 2m). This is because temperate regions have smaller species pools for highly productive habitats since these habitats have been rare in evolutionary history (Pärtel et al., 2007). Similarly, the evolutionary species pool of vegetation in the majority of temperate Eurasian habitats evolved from historically prevalent neutral to slightly high soil pH (Chytrý et al., 2007; Ewald, 2003; Pärtel, 2002).

***2.2 Effects of litter***

Litter cover correlated weakly with the site productivity proxies in our study (i.e., soil humus content and climate gradient, Fig. S2c-d), inconsistent with the idea that litter cover in grasslands is generally considered as the measure of grassland productivity (Grime, 1979). Litter amounts can have profound effects on the structure of plant communities via mechanisms beyond the productivity effects (Facelli & Pickett, 1991; Loydi et al., 2013; Ruprecht et al., 2010; Ruprecht & Szabó, 2012). At optimal ranges, litter cover may provide key benefits for plant communities, such as soil moisture conservation, protection for seed germination and seedlings, and nutrient release during decomposition. Excessive litter amounts may inhibit germination and establishment of plants (e.g., light grass seeds), restrict plant growth (particularly ephemeroids and bulbous plants), and contribute to issues like seed rot and fungal diseases due to excessive moisture retention and increased temperature under the thick layers of compacted or matted litter (Facelli & Pickett, 1991; Ruprecht & Szabó, 2012). Furthermore, litter acts as an abiotic disturbance to grassland plant community (Dembicz, Dengler, et al., 2021; Ruprecht et al., 2010). Thus, the observed by us hump-shaped effects of litter cover on local species richness (Fig. 2q) can be attributed to the *intermediate disturbance hypothesis*, where moderate disturbances reduce interspecific competition, thus promoting species richness, which is largely due to the increased occurrences of rare species.


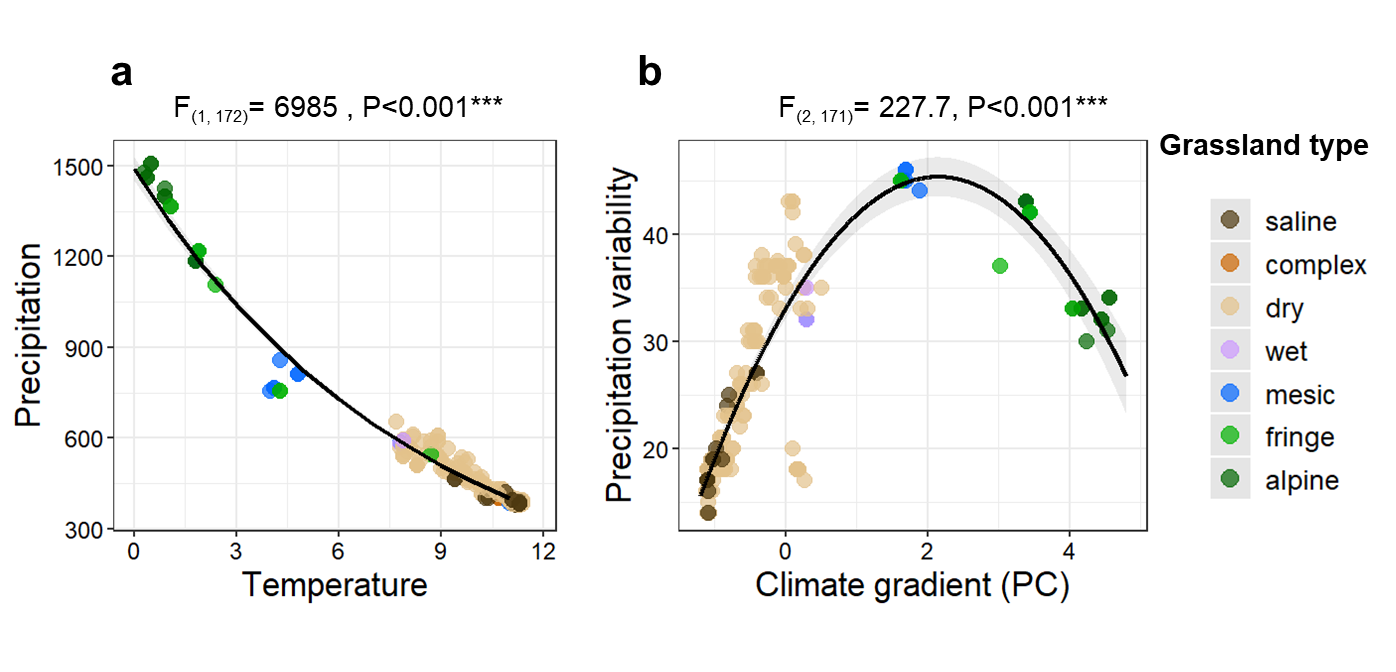


**Fig. S1.** Relationships among annual mean temperature and annual mean precipitation **(a)** and among precipitation variability and climate gradient PC **(b)**. Climate gradient PC is the first principal component derived from the PCA analysis of mean annual temperature and mean annual precipitation and indicates the gradient ranging from hot and dry to cold and wet climatic conditions (see Fig. S11). Precipitation variability is the intraannual precipitation variation, quantified as the standard deviation of the monthly estimates of precipitation from the annual mean. Lines show slopes, and shaded areas around lines show 95% confidence intervals. Results from linear regression models are given above each plot: F-statistic with the degrees of freedom shown in parenthesis and P-value. Colour of data points indicates grassland habitat types.

**
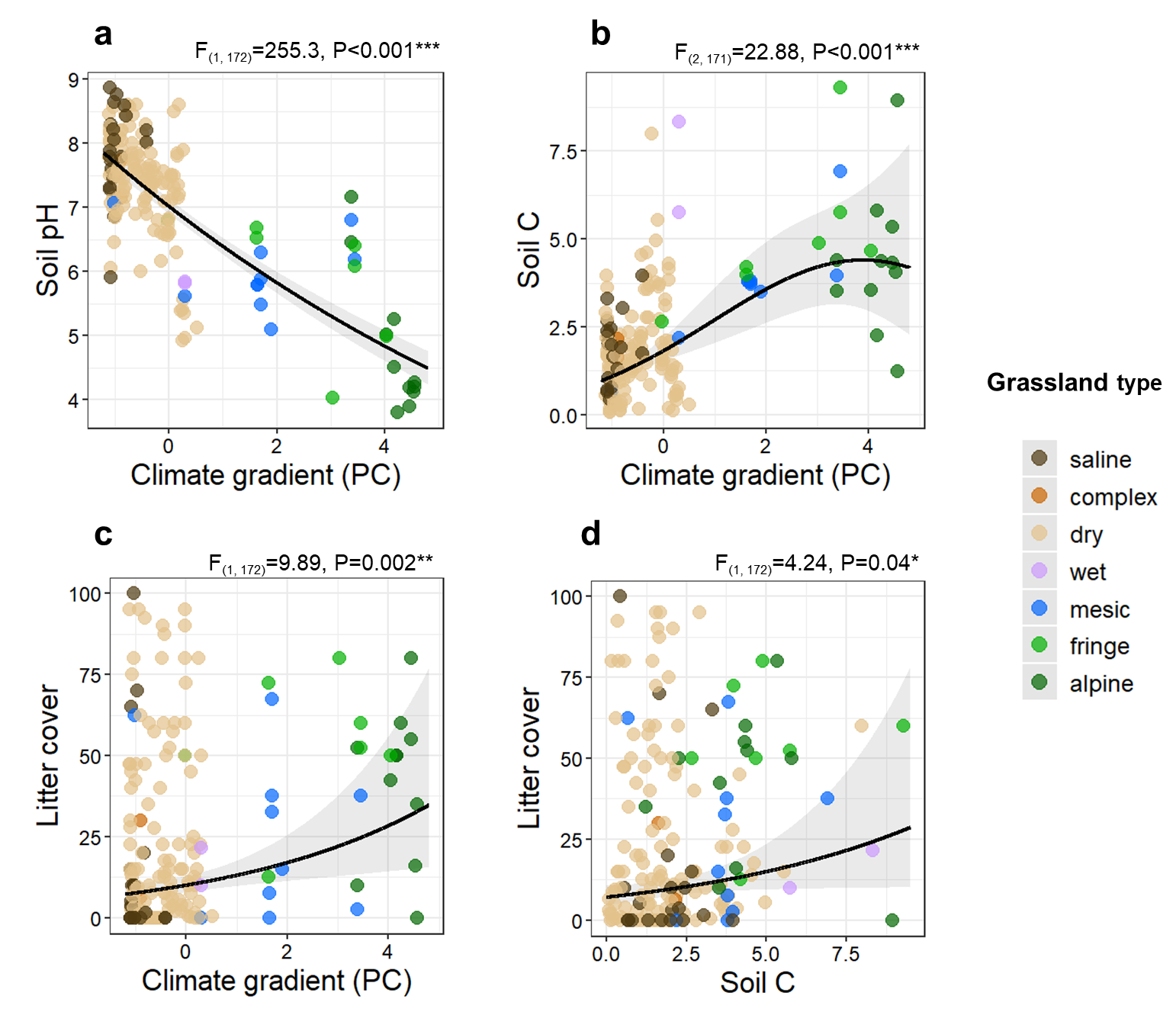
**

**Fig. S2.** Relationships among soil properties and climate gradient PC **(a-b)** and of litter cover with climate gradient PC **(c)** and with soil humus content **(d)**. Climate gradient PC is the first principal component derived from the PCA analysis of mean annual temperature and mean annual precipitation and indicates the gradient ranging from hot and dry to cold and wet climatic conditions (see Fig. S11). Lines show slopes, and shaded areas around lines show 95% confidence intervals. Results from linear regression models are given above each plot and include F-statistic with the degrees of freedom shown in parenthesis and P-value with the following levels of significance: *P≤0.05; **P≤0.01; ***P≤0.001. Colour of data points indicates grassland habitat types.


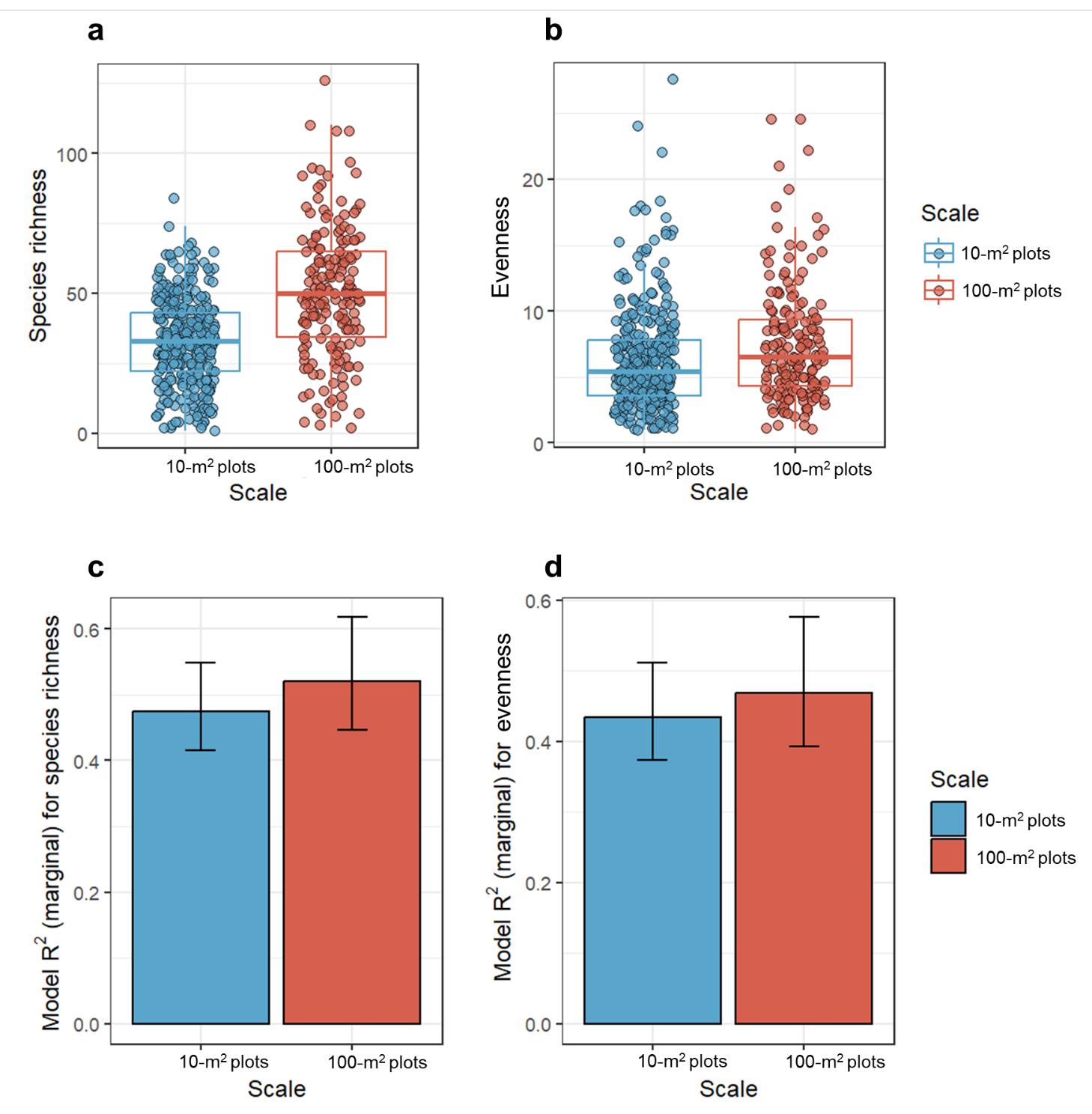


**Fig. S3**. Plant species richness **(a)** and evenness **(b)** for each spatial scale. Boxplots shows minimum, median, and maximum values, while the data points show the values for each plot. Variance explained by the fixed effects (i.e., marginal R^2^) of the mixed models (Table S3) testing species richness **(c)** and evenness **(b)** for each spatial scale. Bars show marginal R^2^ with lower and upper confidence limits. 10-m^2^ and 100-m^2^ scales are indicated by blue and red colours, respectively.

**
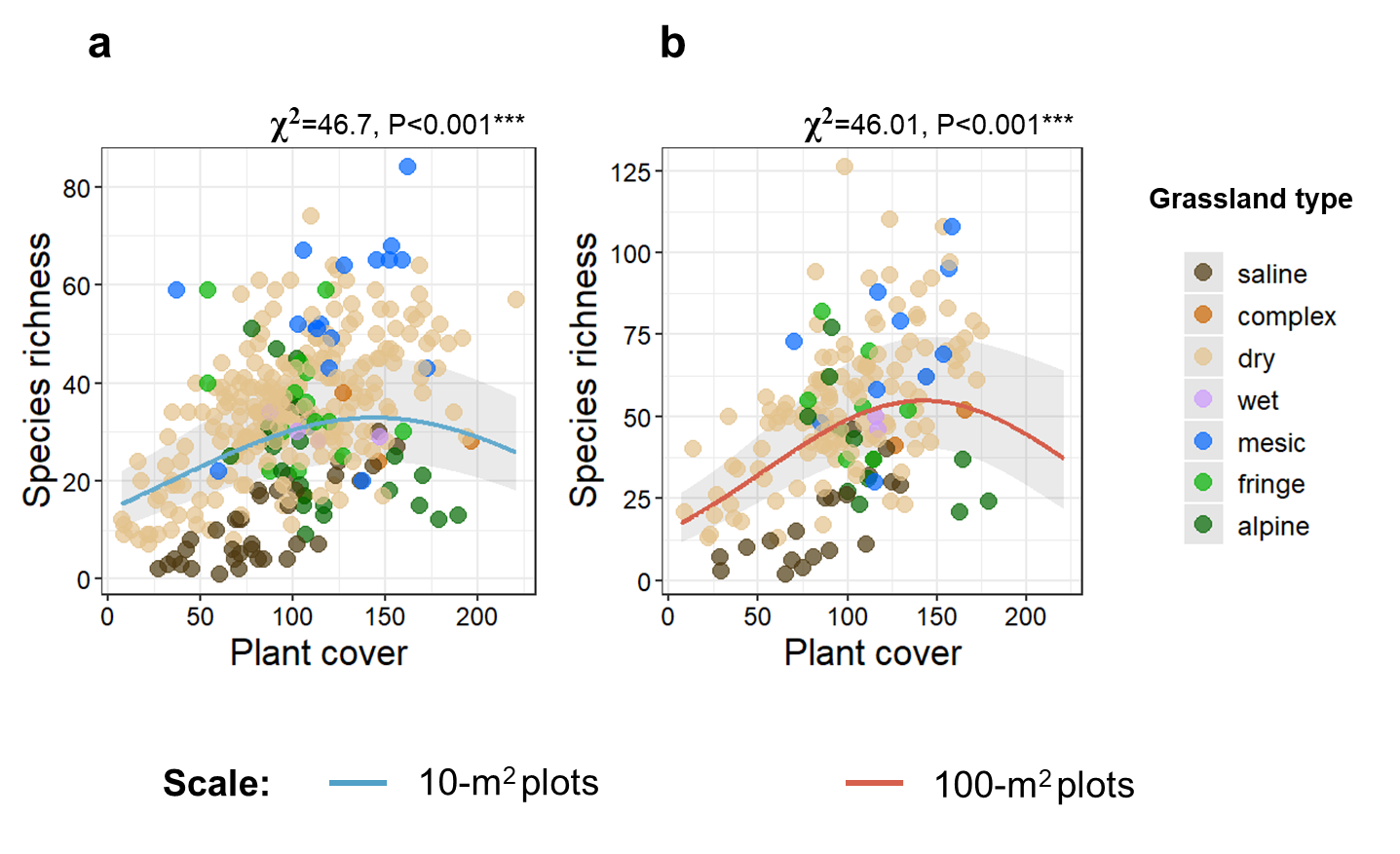
**

**Fig. S4.** Results from mixed models testing the relationship of the cumulative plant cover with local species richness (10-m^2^ and 100-m^2^ plots). Shaded areas around lines show 95% confidence intervals. Colours of data points indicate grassland habitat types.

**
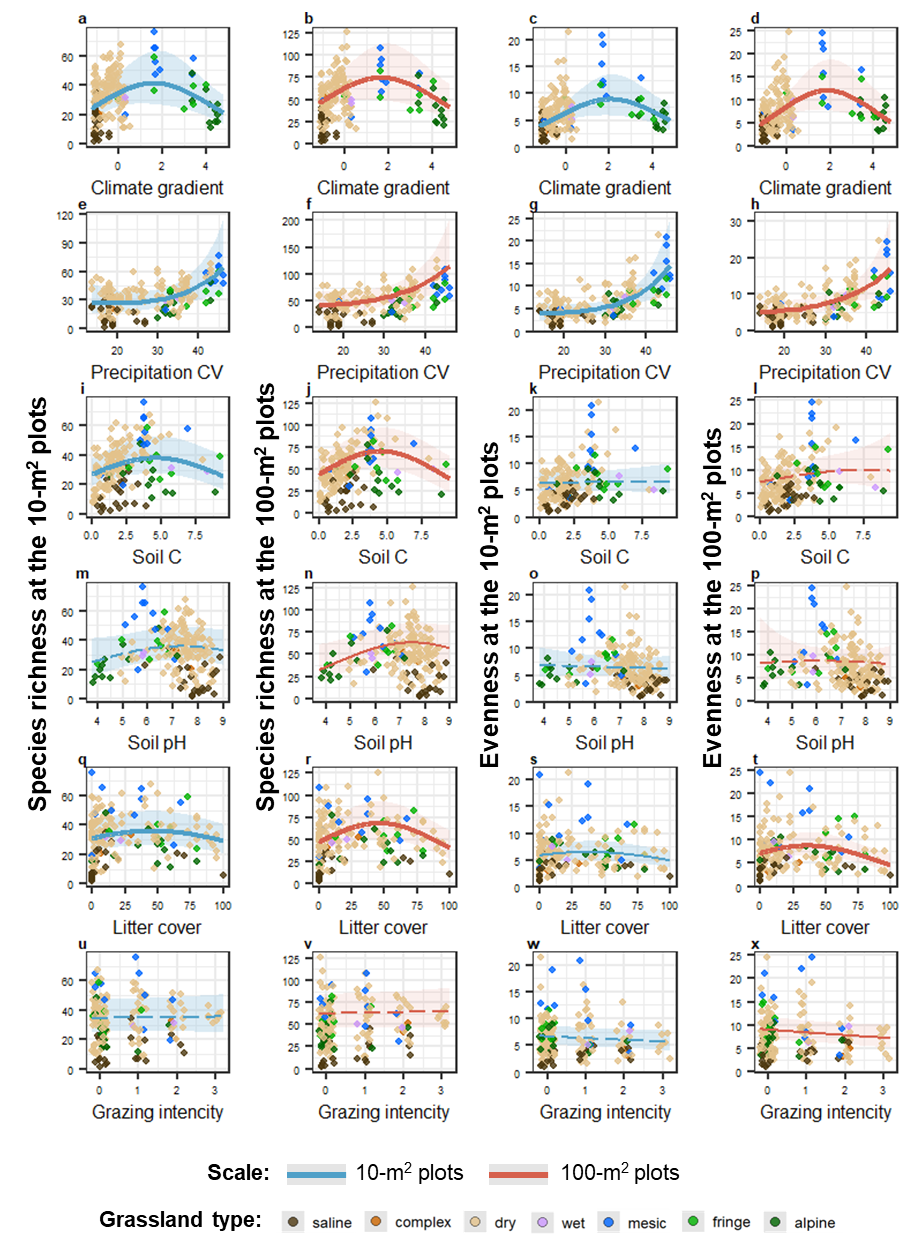
**

**Fig S5.** Results from mixed models testing the effects of environmental drivers on species richness and evenness at the 10-m^2^ and 100-m^2^ plots, shown by blue and red slopes, respectively. For the model results see Table S3. Solid thick lines show significant effects (P<0.05), solid thin lines show marginally significant effects (0.05≤P<0.1), and dashed lines show non-significant effects (P>0.09). Shaded areas around lines show 95% confidence intervals. Colours of data points indicate grassland habitat types.


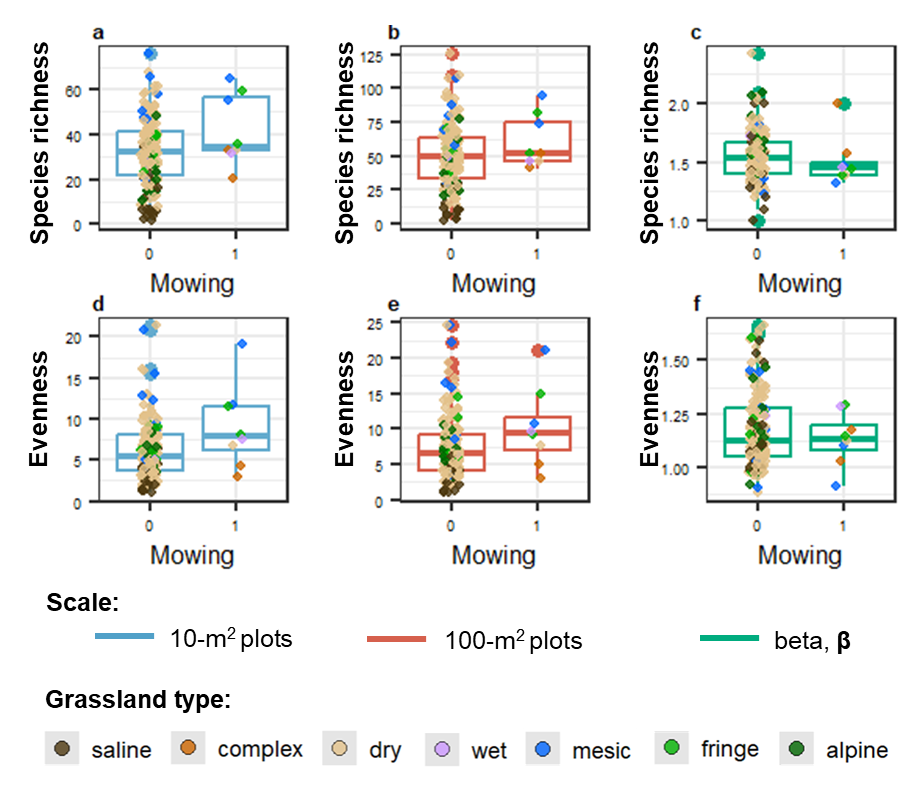


**Fig S6.** Effects of mowing on species richness and evenness at the 10-m^2^ and 100-m^2^ plots (shown by blue and red colours of boxplots respectively) and on **β-**diversity (shown by green boxplots). There were no significant effects of mowing (P<0.05), for the results from the mixed effect models see Table S3 and Table S4. Colours of data points indicate grassland habitat types.


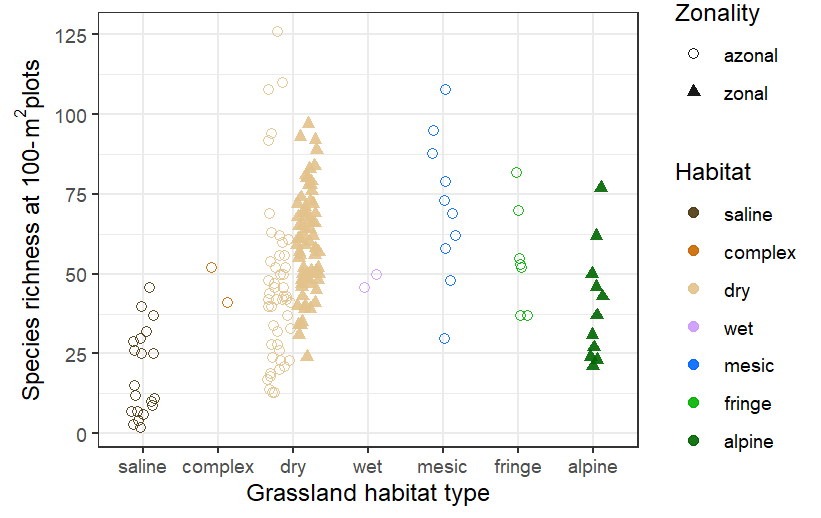


**Fig. S7.**  Plant species richness at the 100-m^2^ plots within each grassland habitat type (indicated by different colours). Points show the values for each plot, where the transparent dots are azonal grassland types and filled triangles are zonal vegetation types.


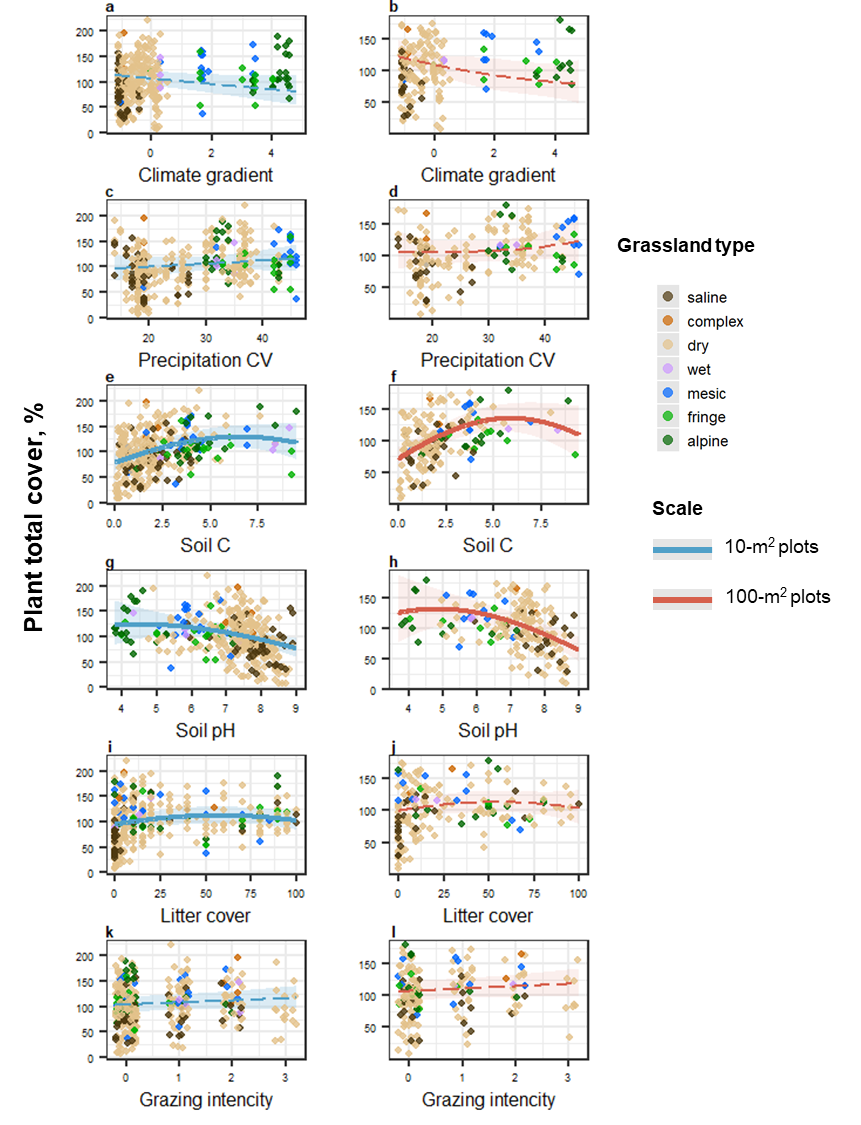


**Fig S8.** Results from mixed models testing the effects of environmental drivers on plant total cover at the 10-m^2^ and 100-m^2^ plots, shown by blue and red slopes, respectively. Solid thick lines show significant effects (P<0.05) and dashed lines show non-significant effects (P≥0.05). Shaded areas around lines show 95% confidence intervals. Colours of data points indicate grassland habitat types.


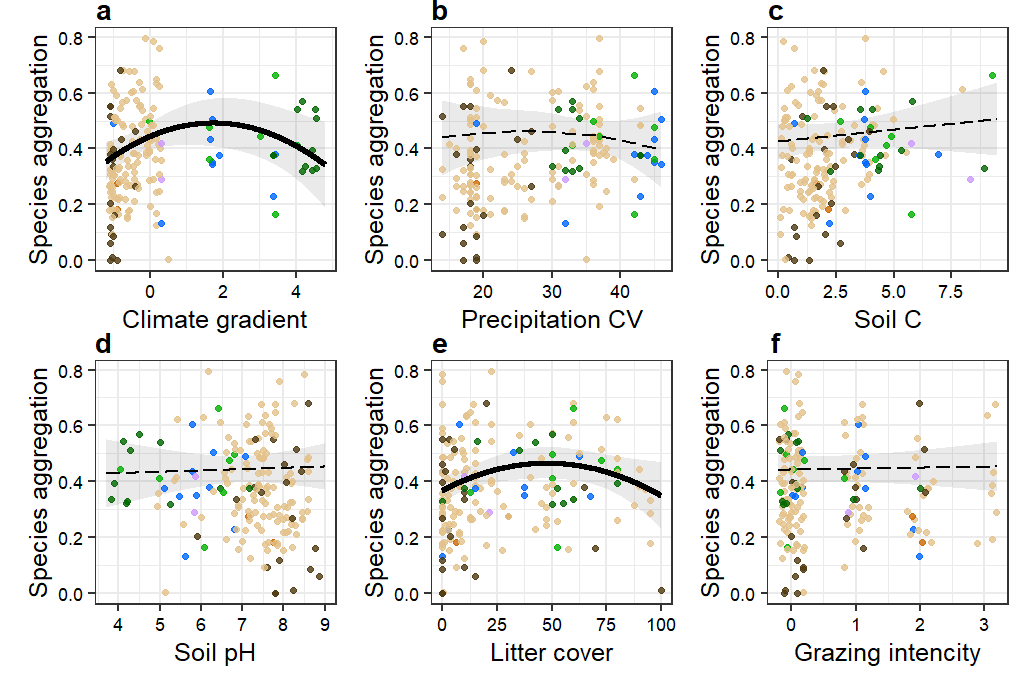


**Fig. S9**. Effects of environmental drivers on intraspecific spatial aggregation. Solid thick lines show significant effects (P<0.05), and dashed lines show non-significant effects (P>0.05). Colours of data points indicate grassland habitat types.


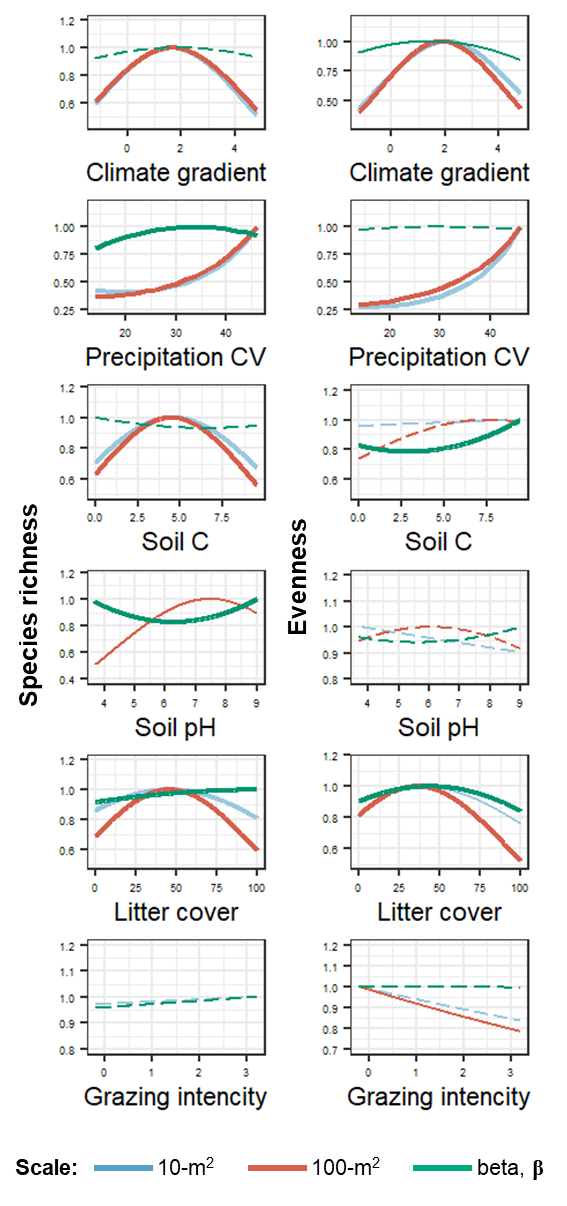


**Fig. S10**. Slopes from mixed models (standardized to the same scale) showing the effects of environmental drivers on species richness and evenness at 10-m^2^ (blue) and 100-m^2^ (red) plots, and on **β**-diversity (green). Model results are detailed in Table S3. Solid thick lines show significant effects (P<0.05), solid thin lines show marginally significant effects (0.05≤P<0.1), and dashed lines show non-significant effects (P>0.09). For raw data visualizations refer to Fig. 2 and Fig S5.


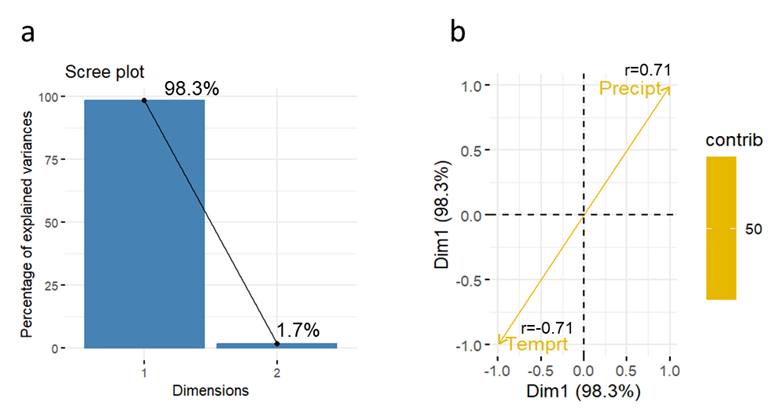


**Fig. S11.** Results of PCA for climate variables. (**a**) Eigenvalues scree plot, showing the percentage of variances explained by each principal component (PC), referred to as dimension. (**b**) The coordinates (i.e., correlations between variables and PC) of climate variables for the PC1 (referred to as Dim1). Both annual precipitation (Precipt) and annual temperature (Temprt) have high and equal contributions to the PC1.

**Table S1.** Summary of published evidence on the **s**cale-dependent effects of plant diversity drivers in grasslands.

| **Scale-dependency of plant-diversity drivers** | **Grassland types** | **References** |
| --- | --- | --- |
| Scale-dependency of climate variables: precipitation and/or temperature | Steppe grasslands in Ukraine,  steppe grasslands in Iran,  dry grasslands in Bulgaria,  Swiss inner-alpine valleys. | (Kuzemko et al., 2016)  (Talebi et al., 2021)  (Dembicz, Velev, et al., 2021)  (Bergauer et al., 2022) |
| Scale-dependency of soil effects: pH, moisture, nutrients, and humus content, has been reported for | Seminatural dry grasslands | (Auestad et al., 2008; Dembicz, Velev, et al., 2021; Turtureanu et al., 2014) |
| Scale-dependency of land use effects | Grazing in pastures in Spain,  mowing in seminatural dry grasslands in Romania,  burning in steppes in Ukraine,  fertilization in various grassland types across North America,  fertilization in the Palaearctic region,  fertilization in grasslands across the globe,  overall land-use intensity in seminatural grasslands in Norway,  overall land-use intensity in seminatural grasslands in Germany,  overall land-use intensity in alpine grasslands across Europe. | (de Bello et al., 2007)  (Turtureanu et al., 2014)  (Kuzemko et al., 2016)  (Chalcraft et al., 2008)  (Dembicz, Dengler, et al., 2021)  (Seabloom et al., 2021)  (Auestad et al., 2008)  (Bolliger et al., 2024)  (Spiegelberger et al., 2006) |

**Table S2.** Overview of the datasets used for the study.

| Dataset ID | Sampling dates | Administrative regions in Ukraine | Latitude range, ° | Longitude range, ° | Altitude range,  m a.s.l. | List of habitat types | List of EUNIS habitat types (3^rd^ level of hierarchy) | List of EUNIS habitat types (3^rd^ level of hierarchy) (Kuzemko et al., 2022; Schaminée et al., 2018) | Number of plots / nested plot series |
| --- | --- | --- | --- | --- | --- | --- | --- | --- | --- |
| 1 | 13-23 July 2010 | Vinnytsia | 48.14 - 48.87 | 27.57 - 29.33 | 78 - 231 | Dry | R1 | R16, R1A, R1B | 42/21 |
| 2 | 15-17 July 2016 | Chernivtsi | 47.74 - 47.81 | 24.89 -25.00 | 968 - 1556 | Mesic | R2 | R21, R22, R23 | 10/5 |
| 3 | 5-8 June 2017 | Kharkiv | 49.84 - 50.37 | 37.12 - 37.76 | 120-161 | Dry | R1 | R15, R1A | 16/8 |
| 4 | 31 May-8 June 2020,  18 July 2020, 24 July 2020 | Mykolaiv, Kherson | 46.64 - 47.49 | 31.39 - 32.03 | 8 - 55 | Dry | R1 | R18, R1B, R1C | 38/19 |
| 5 | 10-13 May 2018,  8 June 2020,  27 June - 8 July 2020 | Mykolaiv | 47.73 - 48.00 | 31.00 - 31.17 | 40 - 79 | Dry | R1 | R16, R1A, R1B | 30/15 |
| 6 | 24 May - 3 June 2021 | Kherson,  Mykolaiv | 46.08 - 47.08 | 31.56 - 34.26 | -4 - 43 | Dry  Mesic  Saline  Complex | R1  R2  R6  X | R11, R18, R1A, R1B, R1C;  R22;  R62;  X36 | 100/50 |
| 7 | 12 - 13 June, 26 June - 5 July, 23 July, 26 July,  6 August 2021 | Kyiv, Vinnytsa, Khmelnytskyi, Ternopil, Chernivtsi, Ivano-Frankivsk | 47.74 - 50.58 | 24.69 - 30.62 | 95 - 1569 | Dry  Mesic  Alpine  Fringe | R1  R2  R4  R5 | R12, R13, R16, R1A, R1P;  R21, R22, R23;  R44;  R51, R55, R56 | 54/27 |
| 8 | 1 - 11 August 2021 | Ivano-Frankivsk, Zakarpatska, Volyn, Rivne | 48.12 - 51.87 | 24.20 - 27.19 | 131 - 1805.2 | Dry  Mesic  Wet  Alpine  Fringe | R1  R2  R3  R4  R5 | R1M, R1P, R1Q;  R21;  R35, R37;  R41, R43, R44;  R56 | 40 / 20 |
| 9 | 9 - 14 September 2021 | Kherson, Mykolaiv | 46.21 - 47.24 | 32.13 - 32.82 | 1 - 103 | Saline | R6 | R63, R64, R65 | 22 / 11 |
| 10 | 24 - 25 June 2022 | Kyiv | 49.94 | 31.09 | 130-140 | Dry | R1 | R1A | 12/6 |
| 11 | 15 - 20 July 2019,  18 - 25 July 2020 | Mykolaiv | 46.80 - 48.12 | 30.07 - 32.23 | 11 - 105 | Dry | R1 | R11, R1B | 18/9 |

**Table S3**. Results of (G)LMMs testing the effects of environmental drivers on local species richness. Model 1 tests the effects of all drivers except the precipitation variability (NA means that the variable was not included in the model). Model 2 tests the effects of the precipitation variability (with all other drivers as covariates), for details see materials and methods in the main text. The column near each predictor shows if quadratic term was used in the model. R^2^m is marginal (for fixed predictors) coefficients of determination. CL is the upper and lower confidence levels for the R^2^m. R^2^c is conditional (for fixed and random predictors) coefficients of determination. Moran’s I statistic tests spatial autocorrelation of residuals for each model: *Obs.I* is the computed Moran's I; *Exp.I*, is the expected value of Moran’s I under the null hypothesis of no spatial autocorrelation; P-value of the test of the null hypothesis, where P < 0.05 indicates significant spatial autocorrelation of residuals.

| **Predictors** | **Species richness at the 10 m^2^ plots** | | | | | | | **Species richness at the 100 m^2^ plots** | | | | | | |
| --- | --- | --- | --- | --- | --- | --- | --- | --- | --- | --- | --- | --- | --- | --- |
|  | Quadratic term for predictors | ***Model 1***  (GLMM) | | | ***Model 2***  (GLMM) | | | Quadratic term for predictors | ***Model 1***  (GLMM) | | | ***Model 2***  (GLMM) | | |
|  |  | **χ^2^** | **Df** | **P-value** | **χ^2^** | **Df** | **P-value** |  | **χ^2^** | **Df** | **P-value** | **χ^2^** | **Df** | **P-value** |
| **Climate gradient (PC)** | poly( , 2) | 7.83 | 2 | **0.02*** | 4.29 | 2 | 0.12 | poly( , 2) | 6.18 | 2 | **0.046*** | 2.24 | 2 | 0.33 |
| **Precipitation variability** | poly( , 2) | NA | NA | NA | 5.38 | 2 | ***0.07*** ’ | poly( , 2) | NA | NA | NA | 6.03 | 2 | **0.049*** |
| **Soil C** | poly( , 2) | 14.12 | 2 | **<0.001***** | 11.28 | 2 | **0.004**** | poly( , 2) | 18.86 | 2 | **<0.001***** | 14.62 | 2 | **<0.001***** |
| **Soil pH** |  | 0.43 | 1 | 0.51 | 0.32 | 1 | 0.57 | poly( , 2) | 4.83 | 2 | ***0.09*** | 5.05 | 2 | ***0.08*** ’ |
| **Litter cover** | poly( , 2) | 8.36 | 2 | **0.02*** | 8.07 | 2 | **0.02*** | poly( , 2) | 20.14 | 2 | **<0.001***** | 20.96 | 2 | **<0.001***** |
| **Grazing intensity** |  | 0.07 | 1 | 0.79 | 0.04 | 1 | 0.84 |  | 0.16 | 1 | 0.69 | 0.05 | 1 | 0.83 |
| **Mowing** |  | 0.01 | 1 | 0.91 | 0.18 | 1 | 0.67 |  | 1.04 | 1 | 0.31 | 1.34 | 1 | 0.25 |
| Model R^2^ | | R^2^m = 0.26 (0.19-0.34); R^2^c = 0.93 | | | R^2^m = 0.48 (0.41-0.55); R^2^c = 0.93 | | | Model R^2^ | R^2^m = 0.34 (0.26-0.46); R^2^c = 0.75 | | | R^2^m = 0.52 (0.44-0.62); R^2^c = 0.79 | | |
| **Residual Spatial**  **Autocorrelation**  **(Moran’s I)** | | Obs.I = -0.001,  Exp.I = -0.003;  sd = 0.002;  P = 0.35 | | | Obs.I = -0.001,  Exp.I = -0.003;  sd = 0.002;  P = 0.49 | | | **Residual Spatial**  **Autocorrelation**  **(Moran’s I)** | Obs.I = -0.003,  Exp.I = -0.006;  sd = 0.004;  P = 0.45 | | | Obs.I = -0.003,  Exp.I = -0.006;  sd = 0.004;  P = 0.49 | | |

**Table S4**. Results of (G)LMMs testing the effects of environmental drivers on evenness at the 10-m^2^ and 100-m^2^ plots. Model 1 tests the effects of all drivers except the precipitation variability (NA means that the variable was not included in the model). Model 2 tests the effects of the precipitation variability (with all other drivers as covariates), for details see materials and methods in the main text. The column near each predictor shows if quadratic term was used in the model. R^2^m is marginal (for fixed predictors) coefficients of determination. CL is the upper and lower confidence levels for the R^2^m. R^2^c is conditional (for fixed and random predictors) coefficients of determination. Moran’s I statistic tests spatial autocorrelation of residuals for each model: *Obs.I* is the computed Moran's I; *Exp.I*, is the expected value of Moran’s I under the null hypothesis of no spatial autocorrelation; P-value of the test of the null hypothesis, where P < 0.05 indicates significant spatial autocorrelation of residuals.

|  | | | | | | | | | | | | | | |
| --- | --- | --- | --- | --- | --- | --- | --- | --- | --- | --- | --- | --- | --- | --- |
| **Predictors** | **Evenness at the 10 m^2^ plots** | | | | | | | **Evenness at the 100 m^2^ plots** | | | | | | |
|  | Quadratic term for predictors | ***Model 1***  (LMM) | | | ***Model 2***  (LMM) | | | Quadratic term for predictors | ***Model 1***  (LMM) | | | ***Model 2***  (LMM) | | |
|  |  | **χ^2^** | **Df** | **P-value** | **χ^2^** | **Df** | **P-value** |  | **χ^2^** | **Df** | **P-value** | **χ^2^** | **Df** | **P-value** |
| **Climate gradient (PC)** | poly( , 2) | 7.41 | 2 | **0.02*** | 0.36 | 2 | 0.84 | poly( , 2) | 9.87 | 2 | **0.01**** | 1.31 | 2 | 0.52 |
| **Precipitation variability** | poly( , 2) | NA | NA | NA | 10.61 | 2 | **0.005**** | poly( , 2) | NA | NA | NA | 7.36 | 2 | **0.03*** |
| **Soil C** | poly( , 2) | 0.04 | 1 | 0.83 | 0.05 | 1 | 0.81 | poly( , 2) | 2.56 | 2 | 0.28 | 1.03 | 2 | 0.60 |
| **Soil pH** |  | 0.19 | 1 | 0.67 | 0.19 | 1 | 0.66 | poly( , 2) | 0.20 | 2 | 0.90 | 0.10 | 2 | 0.95 |
| **Litter cover** | poly( , 2) | 4.92 | 2 | ***0.09*** ’ | 4.20 | 2 | 0.12 | poly( , 2) | 9.75 | 2 | **0.01**** | 9.45 | 2 | **0.01**** |
| **Grazing intensity** |  | 1.94 | 1 | 0.16 | 2.28 | 1 | 0.13 |  | 2.85 | 1 | ***0.09*** ’ | 3.43 | 1 | ***0.06*** ’ |
| **Mowing** |  | 0.14 | 1 | 0.71 | 0.01 | 1 | 0.93 |  | 0.00 | 1 | 0.97 | 0.01 | 1 | 0.92 |
| Model R^2^ | | R^2^m = 0.24 (0.18-0.33); R^2^c = 0.77 | | | R^2^m = 0.43 (0.37-0.51); R^2^c = 0.77 | | | Model R^2^ | R^2^m = 0.32 (0.24-0.45); R^2^c = 0.56 | | | R^2^m = 0.46 (0.39-0.56); R^2^c = 0.56 | | |
| **Residual Spatial**  **Autocorrelation**  **(Moran’s I)** | | Obs.I = -0.0005,  Exp.I = -0.003;  sd = 0.002;  P = 0.27 | | | Obs.I = -0.0007,  Exp.I = -0.003;  sd = 0.002;  P = 0.31 | | | **Residual Spatial**  **Autocorrelation**  **(Moran’s I)** | Obs.I = -0.002,  Exp.I = -0.006;  sd = 0.004;  P = 0.31 | | | Obs.I = -0.002,  Exp.I = -0.006;  sd = 0.004;  P = 0.38 | | |

**Table S5**. Results of LMMs testing the effects of environmental drivers on **β-**species richness (upper panel) and **β-**evenness (lower panel). Model 1 tests the effects of all drivers except the precipitation variability (NA means that the variable was not included in the model). Model 2 tests the effects of the precipitation variability (with all other drivers as covariates), for details see materials and methods in the main text. The column near each predictor shows if quadratic term was used in the model. R^2^m is marginal (for fixed predictors) coefficients of determination. CL is the upper and lower confidence levels for the R^2^m. R^2^c is conditional (for fixed and random predictors) coefficients of determination. Moran’s I statistic tests spatial autocorrelation of residuals for each model: *Obs.I* is the computed Moran's I; *Exp.I*, is the expected value of Moran’s I under the null hypothesis of no spatial autocorrelation; P-value of the test of the null hypothesis, where P < 0.05 indicates significant spatial autocorrelation.

| **Predictors** | **β-species richness** | | | | | | |
| --- | --- | --- | --- | --- | --- | --- | --- |
|  | Quadratic term for predictors | ***Model 1***  ( LMM ) | | | ***Model 2***  ( LMM ) | | |
|  |  | **χ^2^** | **Df** | **P-value** | **χ^2^** | **Df** | **P-value** |
| **Climate gradient (PC)** | poly( , 2) | 1.41 | 2 | 0.50 | 0.00 | 2 | 1.00 |
| **Precipitation variability** | poly( , 2) | NA | NA | NA | 8.91 | 2 | **0.01*** |
| **Soil C** | poly( , 2) | 2.19 | 2 | 0.33 | 2.03 | 2 | 0.36 |
| **Soil pH** | poly( , 2) | 15.53 | 2 | **<0.001***** | 17.00 | 2 | **<0.001***** |
| **Litter cover** | poly( , 2) | 6.79 | 2 | **0.03*** | 4.49 | 2 | 0.11 |
| **Grazing intensity** |  | 1.24 | 1 | 0.27 | 0.20 | 1 | 0.65 |
| **Mowing** |  | 0.06 | 1 | 0.81 | 0.10 | 1 | 0.76 |
| Model R^2^ | | R^2^m = 0.12 (CL: 0.08-0.26); R^2^c = 0.27 | | | R^2^m = 0.2 (CL: 0.14-0.34);  R^2^c = 0.39 | | |
| **Residual Spatial**  **Autocorrelation**  **(Moran’s I)** | | Obs.I = -0.004,  Exp.I = -0.006;  sd = 0.004; P = 0.65 | | | Obs.I = -0.001,  Exp.I = -0.006;  sd = 0.004; P = 0.32 | | |
|  | | | | | | | |
| **Predictors** | **β-evenness** | | | | | | |
|  | Quadratic term for predictors | ***Model 1***  (LMM) | | | ***Model 2***  (LMM) | | |
|  |  | **χ^2^** | **Df** | **P-value** | **χ^2^** | **Df** | **P-value** |
| **Climate gradient (PC)** | poly( , 2) | 5.50 | 2 | ***0.06*** ’ | 2.99 | 2 | 0.22 |
| **Precipitation variability** | poly( , 2) | NA | NA | NA | 0.24 | 2 | 0.89 |
| **Soil C** | poly( , 2) | 10.21 | 2 | **0.01**** | 9.82 | 2 | **0.01**** |
| **Soil pH** | poly( , 2) | 1.14 | 2 | 0.56 | 1.13 | 2 | 0.57 |
| **Litter cover** | poly( , 2) | 11.25 | 2 | **0.004**** | 10.86 | 2 | **0.004**** |
| **Grazing intensity** |  | 0.01 | 1 | 0.92 | 0.05 | 1 | 0.83 |
| **Mowing** |  | 0.97 | 1 | 0.32 | 0.64 | 1 | 0.43 |
| Model R^2^ | | R^2^m = 0.14 (CL: 0.09-0.28);  R^2^c = 0.17 | | | R^2^m = 0.14 (CL: 0.1-0.2);  R^2^c = 0.19 | | |
| **Residual Spatial**  **Autocorrelation**  **(Moran’s I)** | | Obs.I = -0.002,  Exp.I = -0.006;  sd = 0.004; P = 0.39 | | | Obs.I = -0.002,  Exp.I = -0.006;  sd = 0.004; P = 0.35 | | |

**Table S6**. Results of analysis whether precipitation variability adds explanatory power beyond the nonlinear effect of the climate gradient. Model 1 includes both linear and quadratic terms for the climate gradient. Model 2 includes the linear effects of both climate gradient and precipitation variability (i.e., the quadratic term for the climate gradient was replaced by precipitation variability). AIC is Akaike information criterion. ∆AIC shows the absolute difference in AIC between two models. Highlighted in bold when model 2 was better (i.e. AIC was smaller than two units of AIC of the model 1.

| **Model** | **Species richness**  **at the 10-m^2^ plots** | **Species richness**  **at the 100-m^2^ plots** | **β-species richness** |
| --- | --- | --- | --- |
| *Model 1*:  linear and quadratic terms for the climate gradient | AIC = 2454.5 | AIC = 1513.7 | AIC = -53.81 |
| *Model 2*:  quadratic term for the climate gradient is replaced by precipitation variability | AIC = **2452.1** | AIC = **1507.6** | AIC = -39.41 |
| Absolute difference in AIC between two models | ∆AIC = **2.38** | ∆AIC = **6.17** | ∆AIC = 14.39 |
|  | | | |
| **Model** | **Evenness**  **at the 10-m^2^ plots** | **Evenness**  **at the 100-m^2^ plots** | **β-evenness** |
| *Model 1*:  linear and quadratic terms for the climate gradient | AIC = 405.97 | AIC = 243.56 | AIC = -105.53 |
| *Model 2*:  quadratic term for the climate gradient is replaced by precipitation variability | AIC = 417.10 | AIC = 251.90 | AIC = -89.26 |
| Absolute difference in AIC between two models | ∆AIC = 11.1 | ∆AIC = 8.34 | ∆AIC = 16.27 |

**Supplementary references:**

Auestad, I., Rydgren, K., & Økland, R. H. (2008). Scale-dependence of vegetation-environment relationships in semi-natural grasslands. *Journal of Vegetation Science*, *19*(1), 139–148. https://doi.org/10.3170/2007-8-18344

Bergauer, M., Dembicz, I., Boch, S., Willner, W., Babbi, M., Blank-Pachlatko, J., Catalano, C., Cykowska-Marzencka, B., Gehler, J., Guarino, R., Keller, S., Moysiyenko, I., Vynokurov, D., Widmer, S., & Dengler, J. (2022). Scale-dependent patterns and drivers of vascular plant, bryophyte and lichen diversity in dry grasslands of the Swiss inneralpine valleys. *Alpine Botany*, *132*(2), 195–209. https://doi.org/10.1007/s00035-022-00285-y

Bolliger, R., Neuenkamp, L., Prati, D., & Fischer, M. (2024). The effect of land-use intensity on the species-area relationship of plants within temperate grasslands. *In Preparation*, 1–31.

Chalcraft, D. R., Cox, S. B., Clark, C., Cleland, E. E., Suding, K. N., Weiher, E., & Pennington, D. (2008). Scale-dependent responses of plant biodiversity to nitrogen enrichment. *Ecology*, *89*(8), 2165–2171. https://doi.org/10.1890/07-0971.1

Chytrý, M., Danihelka, J., Ermakov, N., Hájek, M., Hájková, P., Kočí, M., Kubešová, S., Lustyk, P., Otýpková, Z., Popov, D., Roleěek, J., Řezníčková, M., Šmarda, P., & Valachovič, M. (2007). Plant species richness in continental southern Siberia: Effects of pH and climate in the context of the species pool hypothesis. *Global Ecology and Biogeography*, *16*(5), 668–678. https://doi.org/10.1111/j.1466-8238.2007.00320.x

Chytrý, M., Tichý, L., & Rolecek, J. (2003). Local and regional patterns of species richness ph/calclum gradient. *Folia Geobotanica*, *38*, 429–442.

de Bello, F., Lepš, J., & Sebastià, M.-T. (2007). Grazing effects on the species-area relationship: Variation along a climatic gradient in NE Spain. *Journal of Vegetation Science*, *18*(1), 25. https://doi.org/10.1658/1100-9233(2007)18[25:geotsr]2.0.co;2

Deák, B., Valkó, O., Török, P., & Tóthmérész, B. (2014). Solonetz meadow vegetation (Beckmannion eruciformis) in East-Hungary - An alliance driven by moisture and salinity. *Tuexenia*, *34*(1), 187–203. https://doi.org/10.14471/2014.34.004

Dembicz, I., Dengler, J., Steinbauer, M. J., Matthews, T. J., Bartha, S., Burrascano, S., Chiarucci, A., Filibeck, G., Gillet, F., Janišová, M., Palpurina, S., Storch, D., Ulrich, W., Aćić, S., Boch, S., Campos, J. A., Cancellieri, L., Carboni, M., Ciaschetti, G., … Biurrun, I. (2021). Fine-grain beta diversity of Palaearctic grassland vegetation. *Journal of Vegetation Science*, *32*, e13045. https://doi.org/10.1111/jvs.13045

Dembicz, I., Velev, N., Boch, S., Janišová, M., Palpurina, S., Pedashenko, H., Vassilev, K., & Dengler, J. (2021). Drivers of plant diversity in Bulgarian dry grasslands vary across spatial scales and functional-taxonomic groups. *Journal of Vegetation Science*, *32*(1). https://doi.org/10.1111/jvs.12935

Facelli, J. M., & Pickett, S. T. A. (1991). Plant litter: its dynamics and effects on plant community structure. *Botanical Review*, *57*(1), 1–32. https://doi.org/10.1007/bf02858763

Grime, J. P. (1979). *Plant strategies, vegetation processes, and ecosystem properties*. John Wiley & Sons, Ltd.

Kuzemko, A., Budzhak, V., Vasheniak, Y., Vynokurov, D., Didukh, Y., Dziuba, T., Iemelianova, S., Kucher, O., Moysienko, I., Tokaryuk, A. I., Khodosovtsev, O. Y., Chorney, I. I., Chusova, O. O., Shapoval, V. V., Shyriaieva, D. V., Balashov, I. O., Brusentsova, N. O., Vasylyuk, O. V., Viter, S. H., … Kuzemko, I. V. (2022). *Atlas of Grassland Habitats of Ukraine. [In Ukrainian]*. Druk Art.

Kuzemko, A., Steinbauer, M. J., Becker, T., Didukh, Y. P., Dolnik, C., Jeschke, M., Naqinezhad, A., Uğurlu, E., Vassilev, K., & Dengler, J. (2016). Patterns and drivers of phytodiversity in steppe grasslands of Central Podolia (Ukraine). *Biodiversity and Conservation*, *25*(12), 2233–2250. https://doi.org/10.1007/s10531-016-1060-7

Loydi, A., Eckstein, R. L., Otte, A., & Donath, T. W. (2013). Effects of litter on seedling establishment in natural and semi-natural grasslands: A meta-analysis. *Journal of Ecology*, *101*(2), 454–464. https://doi.org/10.1111/1365-2745.12033

Palpurina, S., Wagner, V., von Wehrden, H., Hájek, M., Horsák, M., Brinkert, A., Hölzel, N., Wesche, K., Kamp, J., Hájková, P., Danihelka, J., Lustyk, P., Merunková, K., Preislerová, Z., Kočí, M., Kubešová, S., Cherosov, M., Ermakov, N., German, D., … Chytrý, M. (2017). The relationship between plant species richness and soil pH vanishes with increasing aridity across Eurasian dry grasslands. *Global Ecology and Biogeography*, *26*(4), 425–434. https://doi.org/10.1111/geb.12549

Polyakova, M. A., Dembicz, I., Becker, T., Becker, U., Demina, O. N., Ermakov, N., Filibeck, G., Guarino, R., Janišová, M., Jaunatre, R., Kozub, Ł., Steinbauer, M. J., Suzuki, K., & Dengler, J. (2016). Scale- and taxon-dependent patterns of plant diversity in steppes of Khakassia, South Siberia (Russia). *Biodiversity and Conservation*, *25*(12), 2251–2273. https://doi.org/10.1007/s10531-016-1093-y

Ruprecht, E., Enyedi, M. Z., Eckstein, R. L., & Donath, T. W. (2010). Restorative removal of plant litter and vegetation 40 years after abandonment enhances re-emergence of steppe grassland vegetation. *Biological Conservation*, *143*(2), 449–456. https://doi.org/10.1016/j.biocon.2009.11.012

Ruprecht, E., & Szabó, A. (2012). Grass litter is a natural seed trap in long-term undisturbed grassland. *Journal of Vegetation Science*, *23*(3), 495–504. https://doi.org/10.1111/j.1654-1103.2011.01376.x

Schaminée, J. H. J., Hennekens, S. M., Janssen, J. A. M., & Rodwell, J. S. (2018). *Updated crosswalk of the revised EUNIS Habitat Classification with the European vegetation classification and indicator species for the EUNIS grassland , shrubland and forest types*.

Schuster, B., & Diekmann, M. (2003). Changes in species density along the soil pH gradient — evidence from German plant communities. *Folia Geobotanica*, *38*, 367–379.

Seabloom, E. W., Batzer, E., Chase, J. M., Stanley Harpole, W., Adler, P. B., Bagchi, S., Bakker, J. D., Barrio, I. C., Biederman, L., Boughton, E. H., Bugalho, M. N., Caldeira, M. C., Catford, J. A., Daleo, P., Eisenhauer, N., Eskelinen, A., Haider, S., Hallett, L. M., Svala Jónsdóttir, I., … Borer, E. T. (2021). Species loss due to nutrient addition increases with spatial scale in global grasslands. *Ecology Letters*, *24*(10), 2100–2112. https://doi.org/10.1111/ele.13838

Spiegelberger, T., Matthies, D., Müller-Schärer, H., & Schaffner, U. (2006). Scale-dependent effects of land use on plant species richness of mountain grassland in the European Alps. *Ecography*, *29*(4), 541–548. https://doi.org/10.1111/j.0906-7590.2006.04631.x

Srivastava, D. S. S., & Lawton, J. H. (1998). Why more productive sites have more species: an experimental test of theory using tree hole communities. *The American Naturalist*, *152*(4), 510–529. https://doi.org/10.1086/286187

Storch, D., Bohdalková, E., & Okie, J. (2018). The more-individuals hypothesis revisited: the role of community abundance in species richness regulation and the productivity–diversity relationship. *Ecology Letters*, *21*(6), 920–937. https://doi.org/10.1111/ele.12941

Talebi, A., Attar, F., Naqinezhad, A., Dembicz, I., & Dengler, J. (2021). Scale-dependent patterns and drivers of plant diversity in steppe grasslands of the Central Alborz Mts., Iran. *Journal of Vegetation Science*, *32*(2), 1–15. https://doi.org/10.1111/jvs.13005

Turtureanu, P. D., Palpurina, S., Becker, T., Dolnik, C., Ruprecht, E., Sutcliffe, L. M. E., Szabó, A., & Dengler, J. (2014). Scale- and taxon-dependent biodiversity patterns of dry grassland vegetation in Transylvania. *Agriculture, Ecosystems and Environment*, *182*, 15–24. https://doi.org/10.1016/j.agee.2013.10.028

Tyler, G. (2003). Some ecophysiological and historical approaches to species richness and calcicole/calcifuge behaviour - Contribution to a debate. *Folia Geobotanica*, *38*(4), 419–428. https://doi.org/10.1007/BF02803249
